# Design and tests of prospective property predictions for novel antimalarial 2-aminopropylaminoquinolones

**DOI:** 10.1007/s10822-020-00333-x

**Published:** 2020-08-24

**Authors:** Robert D. Clark, Denise N. Morris, Gary Chinigo, Michael S. Lawless, Jacques Prudhomme, Karine G. Le Roch, Maria José Lafuente, Santiago Ferrer, Francisco Javier Gamo, Robert Gadwood, Walter S. Woltosz

**Affiliations:** 1 Simulations Plus, Inc., 42505 10th Street West, Lancaster, CA 93534-7059 USA; 2 Cognigen Corporation, a Simulations Plus Company, Buffalo, NY USA; 3 Kalexsyn, Inc., Kalamazoo, MI USA; 4 Department of Molecular, Cell and Systems Biology, University of California, Riverside, CA USA; 5 Tres Cantos Medicines Development Campus-Diseases of the Developing World, GlaxoSmithKline, Tres Cantos, Madrid, Spain; 6 Present Address: Pfizer Inc., Groton, CT USA

**Keywords:** Antimalarial, ADME, Dihydroorotate dehydrogenase, Drug design, PBPK, QSAR

## Abstract

There is a pressing need to improve the efficiency of drug development, and nowhere is that need more clear than in the case of neglected diseases like malaria. The peculiarities of pyrimidine metabolism in *Plasmodium* species make inhibition of dihydroorotate dehydrogenase (DHODH) an attractive target for antimalarial drug design. By applying a pair of complementary quantitative structure–activity relationships derived for inhibition of a truncated, soluble form of the enzyme from *Plasmodium falciparum *(s-*Pf*DHODH) to data from a large-scale phenotypic screen against cultured parasites, we were able to identify a class of antimalarial leads that inhibit the enzyme and abolish parasite growth in blood culture. Novel analogs extending that class were designed and synthesized with a goal of improving potency as well as the general pharmacokinetic and toxicological profiles. Their synthesis also represented an opportunity to prospectively validate our in silico property predictions. The seven analogs synthesized exhibited physicochemical properties in good agreement with prediction, and five of them were more active against *P. falciparum* growing in blood culture than any of the compounds in the published lead series. The particular analogs prepared did not inhibit s-*Pf*DHODH in vitro, but advanced biological assays indicated that other examples from the class did inhibit intact *Pf*DHODH bound to the mitochondrial membrane. The new analogs, however, killed the parasites by acting through some other, unidentified mechanism 24–48 h before *Pf*DHODH inhibition would be expected to do so.

**Electronic supplementary material:**

The online version of this article (10.1007/s10822-020-00333-x) contains supplementary material, which is available to authorized users.

## Introduction

There were approximately 228 million malaria cases in 2018, with 405,000 deaths attributed to malaria [1]. Infection by *Plasmodium falciparum* and *P. vivax* are the most common causes of the disease in humans, with the former being responsible for the greatest mortality*.* Growing resistance to currently available antimalarial drugs makes identification of new compounds with novel modes of action and activity against resistant parasites a matter of great urgency [2–4].

Finding new molecular targets is one way to reduce the risk of cross-resistance developing after introduction of a new antimalarial. Fortunately, biochemical idiosyncrasies of the parasite provide several distinct targets that reduce the risk of undesirable “off-target” effects. Unfortunately, the expected return on investment for “neglected diseases” like malaria is too low to motivate large-scale commercial development of compounds directed at those disease targets. A few pharmaceutical companies have made substantial contributions to public-sector drug discovery efforts (particularly in the form of screening results), but most work in the area has been done by academic groups and nonprofit organizations.

Funding constraints and limited synthesis resources make in silico and collaborative approaches particularly attractive for such noncommercial applications. Computational methods being employed range from constructing *quantitative* models of the *relationship* of molecular *structure* to the specific biological *activity* of interest (QSARs) or to more general molecular properties related to in vivo absorption, distribution, metabolism, excretion, and toxicity (ADMET QSPRs). *Physiologically based pharmacokinetic* (PBPK) simulations are also potentially useful, in that they are able to combine individual system-specific properties with drug-specific information in order to anticipate how a compound will behave in vivo. Such simulations are needed to take into account complex interactions between physicochemical properties—e.g., lipophilicity, solubility, and permeability. The goal here is to kill the parasites, of course, which makes such modeling applications somewhat different from those in which one is trying to adjust some physiological imbalance. The situation is similar to oncology, though the adversary in the case of malaria is—fortunately—more consistent in its presentation. We were targeting the blood-stage parasite, so the goal was to get compounds to red blood cells in the systemic circulation and keep them there; distribution to peripheral tissues was not particularly desirable.

Efforts to use virtual screening to identify new antimalarials have had limited success. Zhang et al. [5], for example, built QSAR models using training data from a 3,133-compound library that contained 158 confirmed actives. The models obtained were used to screen a commercial database (ChemBridge, San Diego, CA) in order to identify new potential leads. From this virtual screen, 176 compounds representing 22 unique scaffolds were identified and tested for growth inhibition. Of these, 7 were active against *P. falciparum* chloroquine-susceptible (3D7) and chloroquine-resistant (K1) strains ex vivo with a concentration required to kill half of the organisms (XC_50_) of 1 μM or less [5].

Here, we used QSAR models and PBPK simulations to pick an attractive lead series from “hits” in a phenotypic assay, then extended that series by generating a virtual library of novel analogs. An array of predicted properties—*Pf*DHODH inhibition, physiochemical properties, and pharmacokinetic profiles—were used to select a handful of candidates for further evaluation. The full range of predicted properties taken into consideration are spelled out in Supplementary Data Table S2. The ADMET Risks [6] they highlight can reduce oral bioavailability or pose toxicity problems, which makes them relevant in any drug design program. PBPK simulations using predicted ADMET properties as input parameters were carried out to determine whether virtual compounds were likely to have good enough oral absorption and slow enough systemic clearance to sustain a blood concentrations high enough to kill the parasite given a dosing regimen acceptable for existing antimalarial drugs (vide infra). Surviving virtual compounds were synthesized and characterized experimentally.

The methodology described herein focuses on the use of in silico tools for identifying novel lead compounds for synthesis in a way that exploits QSAR and PBPK modeling to minimize the bench resources needed. The workflow can be characterized as follows:

1. Use enzymatic screening data to generate a predictive QSAR model for an attractive target.
2. Apply the QSAR obtained to a large and diverse library of structures and associated phenotypic activity to identify a lead series predicted to exhibit good ADMET properties in aggregate.
3. Create unique combinations of active substituents from discovered scaffolds.
4. Predict activity, ADMET properties, and PK profiles based on QSAR and PBPK models.
5. Choose and synthesize attractive candidates from the analogs identified.
6. Measure biological activities and ADMET properties of the synthesized compounds.
7. Iterate as needed.

The work described here covers a single iteration of this workflow that provided us with an opportunity to assess the ability of our models to make good activity and ADMET property predictions for novel compounds. It also represents a proof-of-concept exercise of our lead discovery and optimization tools and analytical methods developed over the past several years. Our hope was that we might, in the process, also contribute something of value to the search for novel antimalarials.

As a proof-of-concept project, the scale of our work was necessarily limited. The scope was nonetheless broad and deep enough that we encountered several challenges typical of larger drug development projects. Those challenges included needing to revise our original design to incorporate more practical synthesis schemes, to revise synthesis targets to work around failed reactions, and to encountering an off-target mode of action. Fortunately, in our case the off-target activity was one that results in more rapid death of the parasite than inhibiting our intended target does.

## Lead selection and compound design

*Plasmodium* species cannot salvage preformed pyrimidine bases for nucleic acid synthesis as its human hosts can. Dihydroorotate dehydrogenase (DHODH) is a critical enzyme in the de novo pyrimidine synthesis pathway in the parasite and, thus, a potential target for antimalarial drug therapies [7, 8]. The enzyme from *Plasmodium* species is located in the mitochondrion and utilizes ubiquinone (also known as coenzyme Q) as a cosubstrate.

### Predicting DHODH inhibition

Data on *Pf*DHODH inhibition were collected from the literature for a structurally diverse group of compounds. These included triazolopyrimidines [7, 9], diphenylureas [10], and 2-cyano-3-hydroxy acrylanilides [11], among others [12–15]. Quantitative data were not available for inhibition of the intact enzyme, which is bound to the mitochondrial membrane by its hydrophobic *N*-terminus in vivo [16]. A total of 89 of those compounds had been assayed against the soluble recombinant form of *Pf*DHODH (s-*Pf*DHODH) from which the hydrophobic “tail” has been removed. The very low solubility of CoQ-10 limits its utility in vitro, so an artificial ubiquinone (dodecylubiquinone [DQ]) is usually used as substrate instead.

**Fig. 1:**
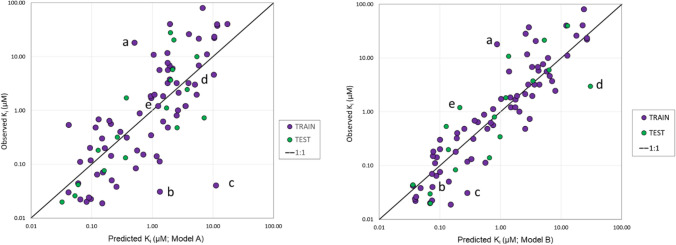
Performance plots for the ANNE regression models developed for in vitro* Pf*DHODH inhibition built from literature data. The plot for Model A is on the left and the plot for Model B is on the right. Labeled points correspond to data from Supplementary Table S1: (a) Tz18; (b) P05; (c) G47; (d) D10; and (e) Tz11

The IC_50_ data for these 89 compounds were converted to inhibition constants (K_i_’s) by assuming competitive inhibition against DQ and applying the method described by Cheng and Prusoff [17]. Two artificial neural network ensemble (ANNE) regression models were constructed (henceforth referred to as Model A and Model B) using ADMET Modeler™ module of ADMET Predictor® [18]. The models were built on distinct 20% hold-out test sets (see Supplementary Methods for details). That and the use of different random number seeds led to somewhat different descriptor sets, complexities and performance statistics (Table 1). The two models complemented each other well, with Model A yielding more conservative activity estimates (fewer false positives) and model B being more sensitive (more prone to generating false negatives). Hence, using them together to identify potent compounds worked better than using either model alone. Plotting the observed versus predicted values from the models illustrated the ability of both models to adequately predict the K_i_ values from both the training set that was used to build the models and the test set used to validate the models (Fig. 1).

**Table 1:** Properties and performance of the *K*_i_ models

Statistic	Model A (1 × 9^a^)			Model B (2 × 29)		
	Train^b^	Verify^c^	Test^d^	Train	Verify	Test
MAE	0.51	0.52	0.44	0.29	0.37	0.43
SRCC	0.77	0.78	0.76	0.92	0.88	0.88
Q^2^	–	0.83	0.68	–	0.90	0.71

*RMSE* root mean square error, *MAE* mean absolute error, *SRCC* Spearman’s rank correlation coefficient, *Q*^*2*^ predictive relevance for the verification and test sets

^a^ANN architecture indicated by number of neurons x number of input descriptors

^b^Training set

^c^Internal test set used trigger early stopping

^d^Hold out test set [6]

### Selecting a lead series

The developed QSAR models were used to analyze structures for 13,533 antimalarials identified in a phenotypic screen of nearly 2 million compounds that was carried out by GlaxoSmithKline (GSK). The published data included *P. falciparum* growth inhibition for the chloroquine-sensitive 3D7 (PubChem AID 2306) as well as for the multidrug resistant strain Dd2 (PubChem AID 2302). Data from a counter screen for mammalian cytotoxicity was available from PubChem AID 2303 [19].

There were some important limitations in working with this data set. First, evaluating efficacy against parasites cultured in human erythrocytes provides no direct information on a compound’s mode of action. Secondly, only results for the active compounds (those that inhibited parasite growth by at least 80% at a concentration of 2 μM) were reported. The lack of data on inactives posed a considerable risk of synthesis resources being wasted on seemingly “novel” analogs that in fact had already been synthesized but not reported due to their lack of activity. Lastly, many of the compounds in the screening data set exhibited undesirable physical properties and/or undesirable substructures (e.g., multiple halogenated thiophene rings) that would cause them to be avoided by many pharmaceutical companies [20].

Candidate lead series from the active compounds in the phenotypic screening data set were identified and structural classes were generated in MedChem Studio™ [21] based on shared substructures. Unlike most other approaches [22], the method used in MedChem Studio does not cluster compounds (in this case, actives) into exclusive subsets based on the degree of pairwise overlap in their substructural fingerprints. Rather, it generates nonexclusive classes of molecules that share a maximal common substructure. In most cases, this shared substructure is, itself, a scaffold in the medicinal chemistry sense. If not, it can readily be modified to become one. The nonexclusivity is advantageous for lead series identification because it is typically easier to see cases where overlapping classes can profitably be combined by merging the scaffolds that define them.

Classes were evaluated as potential lead series based on the QSAR-predicted inhibitory potencies against *Pf*DHODH and the degree of parasite growth inhibition observed in the phenotypic screen for chloroquine-sensitive *P. falciparum* 3D7 (PubChem AID 2306). ADMET Risk™, which indicates how many of an array of ADMET property rules are violated by a compound [6], was also taken into consideration. The rule thresholds are calibrated against a subset of the World Drug Index (WDI) that is enriched in orally delivered commercial drugs similar to that used by Lipinski, et al., for anticipating solubility and oral absorption problems [23]. Ninety percent of the WDI reference set have an ADMET Risk score less than seven.

Low group averages were preferred for both predicted K_i_ and ADMET Risk, but the spread in each profile was also important. Lack of variation in potency indicates a “lack of SAR”, which makes it difficult to enhance activity against the target by modifying structure. Similarly, a lack of variation in ADMET Risk suggests that it will be difficult to engineer out potential liabilities by changing structures. The exception would be a class wherein all ADMET Risk scores are very low, and that was never the case in this dataset.

**Fig. 2:**
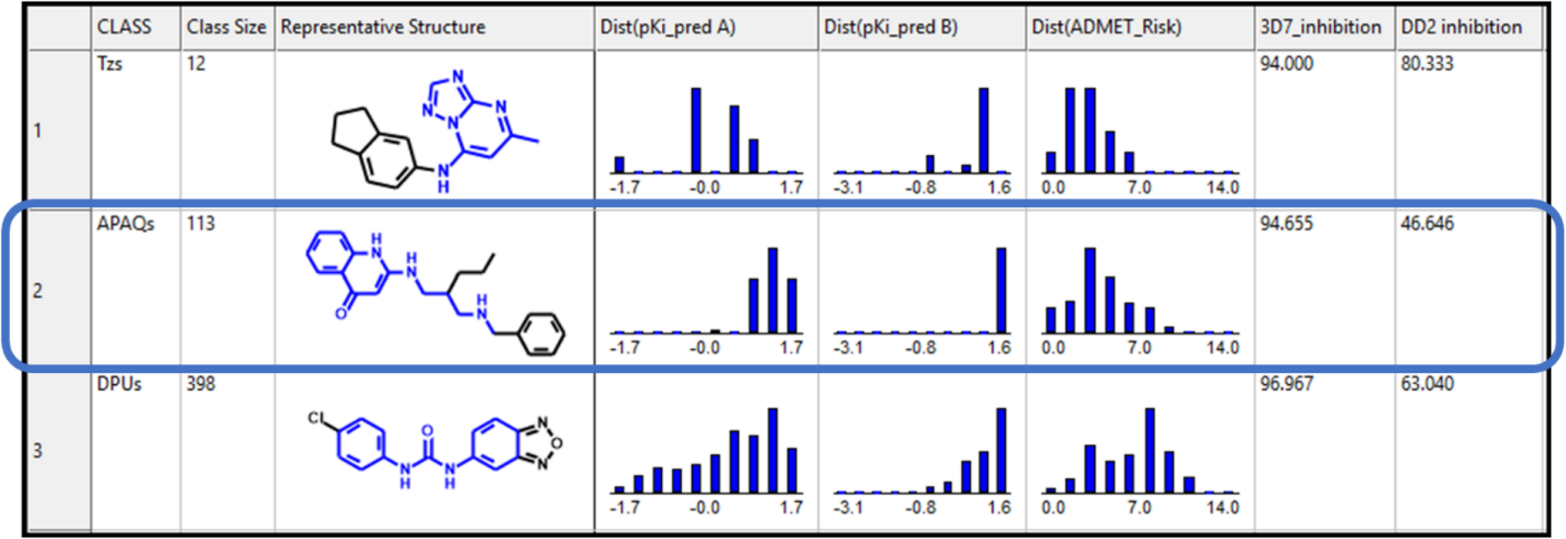
Comparing property distributions across three representative classes: triazolopyrimidines (Tzs), aminopropylaminoquinolones (APAQs) and diphenylureas (DPUs). The portion of each representative structure highlighted in blue corresponds to the class scaffold. Growth inhibition is shown as %inhibition vs. *P. falciparum* strains 3D7 and DD2, which are chloroquine-sensitive and -resistant, respectively. pKi_pred values are the negative log of the predicted K_i_ in µM, so a larger number indicates higher potency; their distribution for Models A and B are shown. ADMET Risk is a measure of likely development liabilities that can range from 0 to 24 [6]

Results for three classes at an intermediate stage of the series definition process are shown in Fig. 2. Two of the classes—diphenyl ureas (DPUs) and triazolopyrimidines (Tzs)—represent *Pf*DHODH inhibitor lead series that had already been explored or were being actively explored by other research groups [24, 27].

**Fig. 3:**
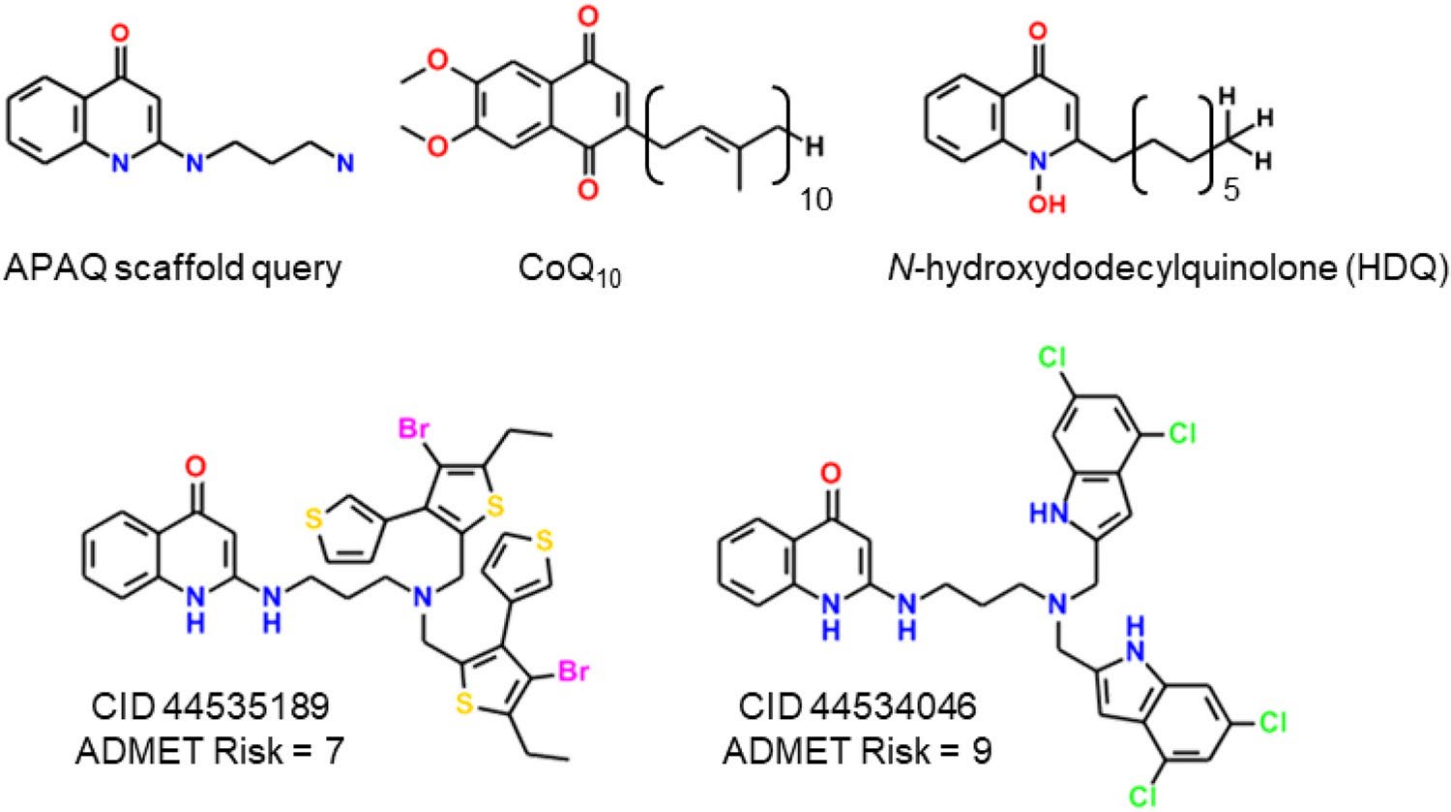
Scaffold and structures of compounds associated with the APAQ lead series

The 2-(3-aminopropylamino)-4-quinolone (APAQ) class was particularly promising because its scaffold bore a distinct resemblance to ubiquinone, which is a cosubstrate for *Pf*DHODH in vivo (Fig. 3). They also resembled *N*-hydroxy-2-dodecyl-4-quinolone (HDQ) (Fig. 3) which Dong et al*.* had determined to have good antimalarial activity, with *Pf*DHODH as its primary target [25]. Although they reported that HDQ inhibited parasite growth in culture and were able to show that *Pf*DHODH was the site of action, they did not have access to soluble enzyme. Hence the XC_50_ obtained in culture could not be translated into an in vitro K_i_ for the enzyme. HDQ was therefore not included in the data set used to build the *Pf*DHODH inhibition models. Two quinolones found among the “hits” reported by Patel et al. [8] were initially grouped with some of the APAQs. These lack the defining 2-amino substitution and so did not appear in the final lead series, which was comprised of 113 actives.

None of the APAQs were reported to be cytotoxic to the mammalian HepG2 cells used in the companion AID 2303 screen, which suggests that the intrinsic risk of mammalian toxicity is small. That said, many of them had discouragingly high ADMET Risk scores. In particular, the two most active examples (Fig. 3) violated 7 and 9 of the standard ADMET Risk rules, respectively. Both are large and were predicted to be excessively hydrophobic, to be highly bound in plasma, to exhibit low water solubility, and to be acutely toxic to rats. In addition, CID 44535189 has an excessively large number of rotatable bonds and was predicted to exhibit chronic toxicity in mice. Besides the five likely liabilities shared with CID 44535189, CID 44534046 was predicted to be metabolized by CYP2D6 and CYP3A4, to inhibit CYP3A4, and to bind to the *human ether-a-go-go* (*hERG*) gene product.

### Analog generation and selection

The predictions of high lipophilicity, low water solubility, high susceptibility to metabolism by cytochrome P450s (especially CYP2D6), and hepatotoxicity were common among the actives in the APAQ class. Substituents from the less risky APAQs were combinatorially reshuffled in silico to generate lead candidates with more favorable activity and property profiles. Substituents from each R-group were drawn from the known actives in the class. Doing so increases the likelihood that the structures produced will be synthetically accessible, since at least one example already exists where that substituent was placed at that position. In addition: only single substitutions were allowed at the distal nitrogen; only simple substituents (hydrogen, halogen, methyl, or trifluoromethyl) were allowed on the quinolone ring; and substitution was restricted on the distal aromatic rings. The resulting constrained combinatorial R-group explosion produced a virtual library of ~ 99,000 novel analogs.

Attractive lead analogs from the virtual library were selected based on their predicted *Pf*DHODH inhibitory power in both QSAR models, their predicted ADMET properties, and their expected ease of synthesis. Compounds for which the predicted *Pf*DHODH K_i_ for Model A was greater than 0.1 µM and with ADMET Risk > 4 were set aside, as were compounds that were out-of-scope^1^ with respect to Model A. Though Model B was useful for identifying good lead series, it failed to provide much further discrimination between APAQ analogs, hence whether its prediction for a particular molecule was out-of-scope or not was disregarded.

As is often the case, descriptors for some of the compounds ultimately selected for synthesis based on activity predictions fell well outside the range of descriptors for compounds used to train one or more of the metabolism models available at the time. Predictions for such compounds were considered “out-of-scope” extrapolations and, therefore, not necessarily reliable. Compounds with in-scope predictions were favored in selecting candidates for further analysis but ignoring those for which *any* prediction was out-of-scope was impractical. Such limitations in coverage are common for any novel chemistry, but they are also a major motivation for continuously refining and expanding ADMET models to improve their predictive performance.

Once analogs predicted to have relatively low potency or discouraging ADMET properties had been filtered out, approximately 34,001 analogs still remained. Of those, 17,334 bore symmetrical central diamine moieties, a feature expected to greatly simplify synthesis. ADMET Predictor now includes a synthetic difficulty score analogous to that described to the synthetic accessibility score described by Ertl and Schuffenhauer [26]. That functionality was not available when this project began, however, so assessment was carried out manually by visual inspection of analogs displayed in a tile view, i.e., in a gridded rather than a row format. In retrospect, the manual process was reasonably effective. The synthetic difficulty scores for the symmetrical diamine analogs ranged from 1.95 to 5.0 (median 3.62, where 10 is most difficult) and the final dozen candidates (vide infra) ranged from 2.5 to 4.15 (median 3.35), indicating that the final candidates were fairly representative of the initial pool in terms of synthetic accessibility.

Marginal aqueous solubility was a central concern that had to be balanced against model predictions that substitution at the central carbon of a 1,3-diaminopropyl bridge should improve activity and reduce CYP metabolism. This and other ADMET Risks prompted us to manually expand the library by introducing small point changes to some marginal analogs. One such change was “mutating” a *gem*-cyclohexyl ring at the central carbon of the aminopropylamino bridge to a *gem*-cyclopentyl ring. No such compound existed among the GSK actives, though there were several derived from *trans* 1-amino-2-aminomethylcyclopentane [19, 27]. A 1,2-ring is expected to adopt a quite different 3D conformation from that for a 2,2-ring, thereby placing the terminal group at quite different positions in space.

After the initial set of lead compounds were selected, their expected pharmacokinetic profiles were evaluated by applying the in silico predictions and standard human physiologies in GastroPlus® [28] to predict plasma concentration-versus-time profiles. Property estimates were taken from ADMET Predictor and dosing regimens were similar to those used for existing antimalarial drugs, e.g., chloroquine. Target plasma concentrations were based on predicted K_i_ values.

**Fig. 4:**
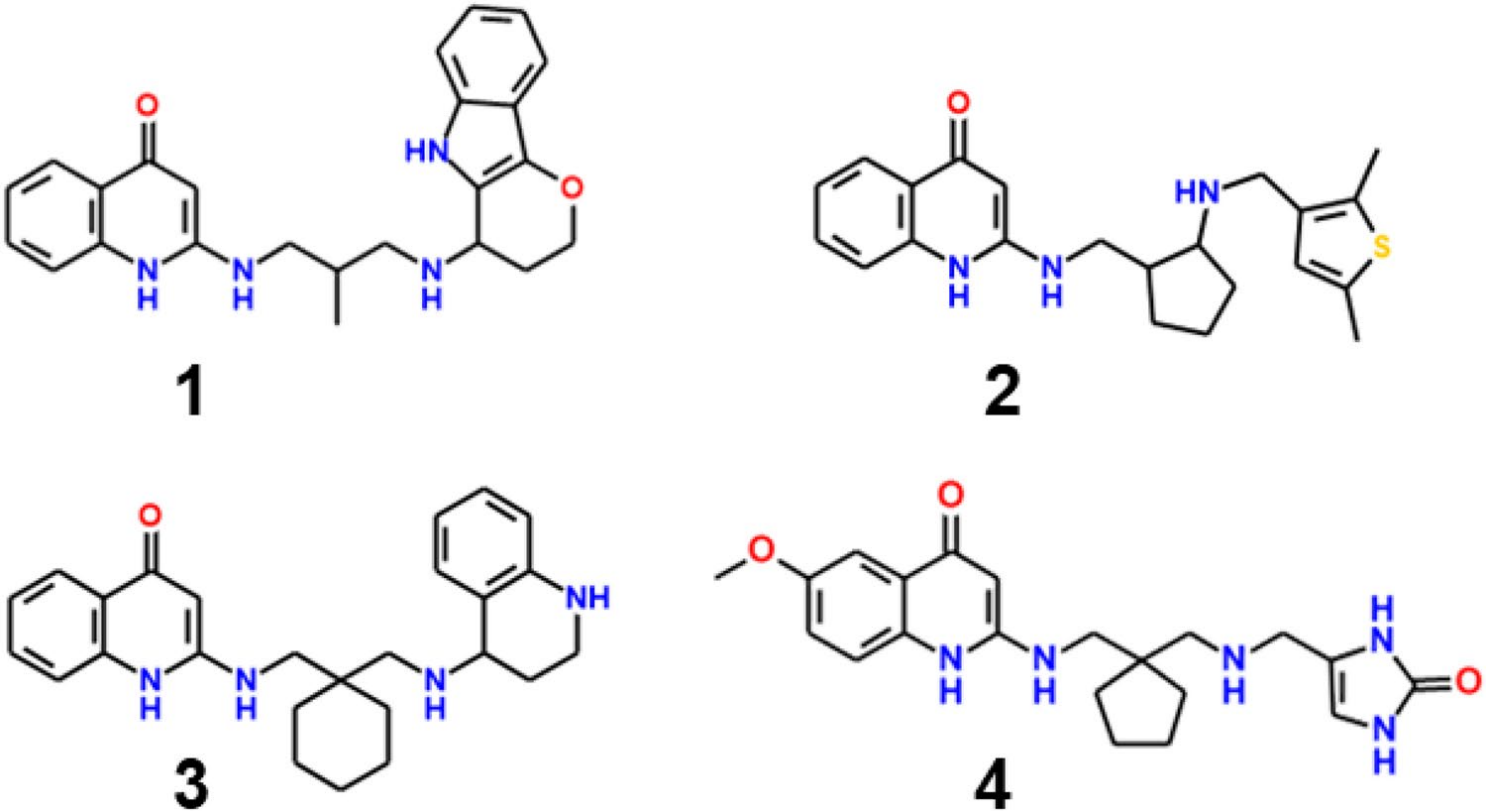
Four of the 12 APAQ synthesis targets initially put out for bids

An initial set of diverse synthesis candidates (Supplementary Table S2) was selected from among those predicted to have good potency as well as an acceptable ADMET Risk score and favorable simulated in vivo PK profiles. The twelve synthesis targets produced by this process had structural variations at three different positions: the R1 substituent on the quinolone end of the scaffold; the R2 and R3 substituents off of the distal nitrogen of the aminopropylamino bridge; and the R4 and R5 substituents off of the central carbon in the bridge. The beginning of the reduction-to-practice phase was announced in a press release in September 2011 [29] and bids were solicited from several contract synthesis companies. Four of the targets distributed are shown in Fig. 4.

**Fig. 5:**
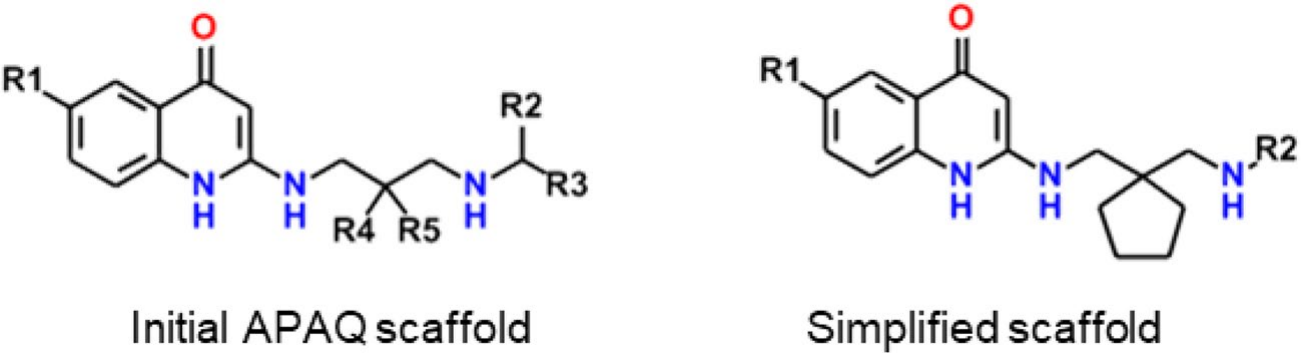
Initial scaffold used for R-Group explosion versus the final, simplified scaffold shared by the analogs that were synthesized

The individual compounds proposed were synthetically feasible, but the number of separate intermediates and variations in reaction conditions required would have been prohibitively expensive. Instead, variations were restricted to either end of the molecule and we focused on a single kind of aminopropylamino bridge. Doing so made it likely that similar reaction conditions and intermediates could be used to make multiple analogs. The convergent synthesis plan greatly reduced the potential for complications and budget overruns. Though not often dwelt on in the literature, such considerations are often a critical practical consideration for any molecular design project; hence their explicit inclusion here. In the end, we settled on seven analogs built around a simplified scaffold bearing the novel gem-cyclopentyl group at the central carbon of the bridge. Diversity was provided by placing three different substituents on the distal nitrogen (i.e., R2) (Fig. 5).

Kalexsyn, Inc. (Kalamazoo, MI) synthesized a revised list of seven target analogs. Carbonyl precursors suitable for reductive amination were available or accessible for some of the desired R2 substituents, considerably simplifying their synthesis.

## Experimental results and discussion

The candidates chosen under the plan outlined above struck a reasonable balance between structural diversity and being accessible via reasonably convergent and parallel synthetic methodologies. Despite our hopes and best intentions, however, the particular substituents targeted for the distal nitrogen still needed some tweaking as regards how the coupling was carried out. Similarly, although the 6-methoxy analog moiety is very similar in structure to that in the 6-chloro and unsubstituted quinolones, its synthesis required a distinct approach. Fortunately, both approaches were relatively straightforward. What is provided here is an overview for illustrative purposes. That said, it is complete enough to give a sense of how much effort was involved in generating the seven targeted analogs. More details are provided in the Supplementary Materials.

### Synthesis

**Scheme 1:**
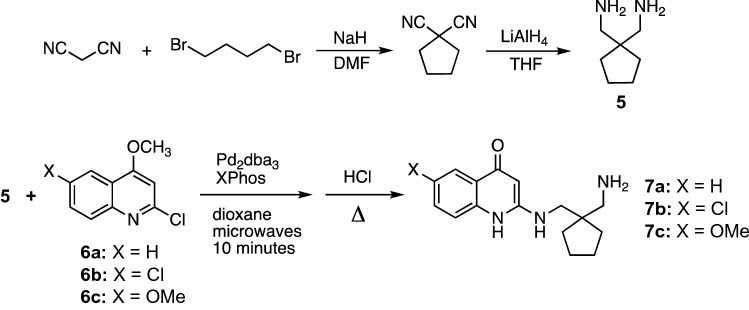
Syntheses of three variations on the free amino APAQ scaffold **7**

The general synthesis procedure used is outlined in Scheme 1. *gem*-Dicyanocyclopentane was prepared by condensation of malononitrile with dibromobutane and reduced to the diamine **5** using lithium hydride in tetrahydrofuran. Subsequent reaction with 2-chloro-4-methoxyquinolines yielded **7a–c** after hydrolysis in HCl.

**Scheme 2:**
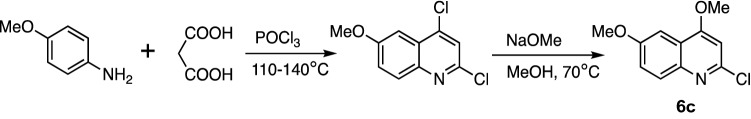
Synthesis of 2-chloro-4,6-dimethoxy quinoline

2-Chloro-4,6-dimethoxyquinoline (**6c**) was prepared by condensation of malonic acid with *p*-anisidine, yielding 2,4-dichloro-6-methoxy quinoline [30], which was converted to the desired dimethoxy compound by treatment with methoxide in methanol (Scheme 2).

**Scheme 3:**
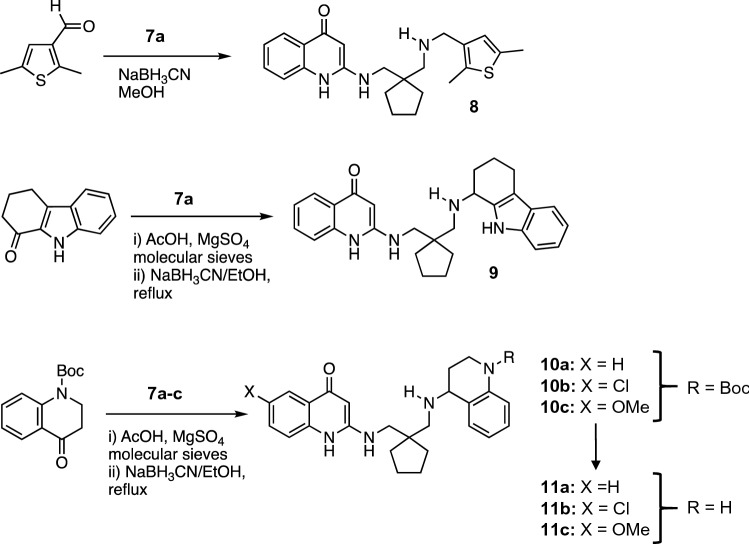
Synthesis of targeted APAQs **8–11**

Reductive alkylation of the distal amino group in **7a** with thiophene carbaldehyde to yield **8** proceeded readily with cyanoborohydride in methanol at room temperature, whereas preliminary formation of the imine and heating were required to generate the tetrahydrocarbazole analog **9** and the tetrahydroquinolines **10a–c** from the corresponding ketones (Scheme 3).

Protection of the indole nitrogen in the former case was not necessary. In fact, difficulties with deprotecting the product made it counterproductive. Blocking the amino group in the tetrahydroquinolone, on the other hand, was essential. Though reductive amination with the free amine failed, the t-butyloxycarbonyl(Boc)-protected dihydroquinolone reacted cleanly. Deprotection with ZnBr_2_ in dichloromethane (DCM) or transesterification in formic acid followed by hydrolysis in NaOH afforded the targeted analogs **11a–c** (Scheme 3).

**Scheme 4:**
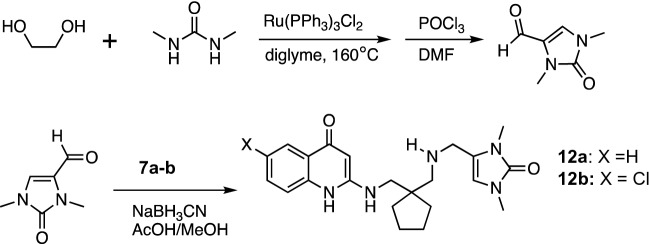
Synthesis of imidazolinone analogs **12a** and **12b**

Analogs bearing an unsubstituted imidazolinone ring in place of the dimethylthiophene ring in **8** were attractive in terms of predicted activity and ADMET properties. Unfortunately, several attempts to prepare and purify them failed. Some *N*-protected intermediates were generated, but all broke down during deprotection or during purification. The 1,3-dimethylimidazolinones **12a** and **12b** were synthesized in their stead (Scheme 4). The added methyl groups were predicted to reduce solubility somewhat, but absorption was expected to be enhanced. Moreover, the methyl groups were predicted to be cleaved off in the liver to produce the original targets. This change in targets necessitated the use of *N,N’*-dimethylimidazolinone-4-carboxaldehyde, which was prepared from ethylene glycol and *N*,*N*’-dimethyl urea, then formylated with POCl_3_ in dimethylformamide. Condensation with the respective free amino precursor yielded the desired APAQs (Scheme 4).

### ADMET properties: predicted vs. experimental

To become a drug candidate, a lead molecule must possess many favorable pharmacological properties, including good solubility, metabolic stability, and low toxicity as well as activity. Hence, ADMET QSARs and QSPRs provide a valuable complement to activity models in avoiding unfavorable ADMET profiles, provided the predictions they provide are accurate and reliable. Measured physicochemical properties of the most active synthesized compounds were determined experimentally and are compared with the predictions from ADMET Predictor 9.0 in Table 2. Most of the observed and predicted properties have a root mean squared error (RMSE) of 0.8 log units or less. Aqueous solubility was an exception, but not an unexpected one. That is because the compounds were all isolated as foams that subsequently solidified to glassy solids, the solubilities of which can be quite difficult to measure accurately.

**Table 2:**
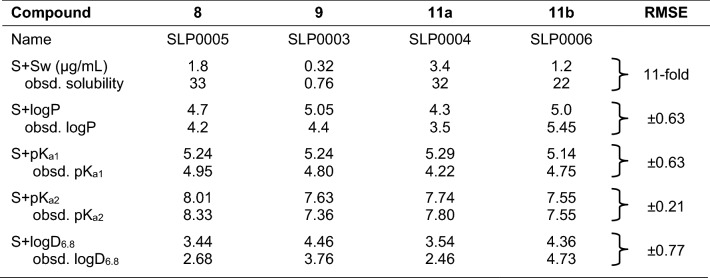
Predicted and measured physicochemical properties of the candidates

*logP* logarithm of the octanol:water partition coefficient, *logD*_*6.8*_ logP at pH 6.8, *obsd* observed, *pK*_*a1*_ negative logarithm of the first dissociation constant, *pK*_*a2*_ negative logarithm of second dissociation constant, *RMSE*, root mean squared error, *S+* denotes a proprietary model from Simulations Plus, Inc., *Sw* aqueous solubility

Rates of in vitro clearance determined from a panel of recombinant microsomes containing human CYPs were measured, as was that by human liver microsomes (HLMs). The results obtained are shown in Table 3. The overall performance of the models in ADMET Predictor is good, but interpretation is more complicated than for the physicochemical properties. These are not predictions of simple intrinsic clearance, as several of the relevant concentrations at half-maximal metabolic velocity (K_m_’s) are predicted to be low enough for partial saturation to be an issue (Supplementary Table S3). This underscores the desirability of having affinity models (here, for K_m_) available to put data on microsomal stability obtained at a single concentration into proper context.

**Table 3:**
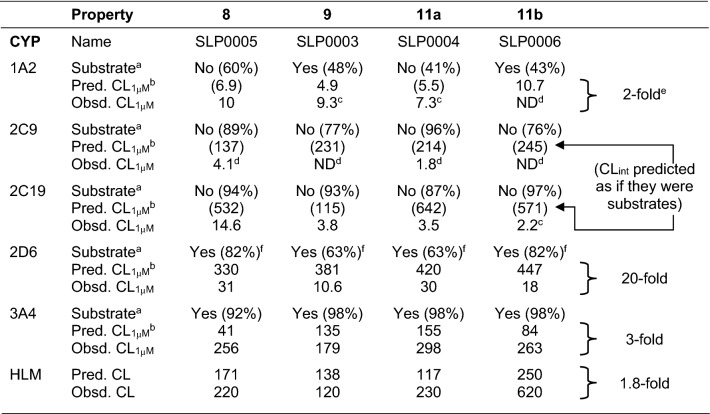
Predicted and measured rates of metabolism by CYPs in vitro

*CL*_*int*_ intrinsic clearance, *CYP* cytochrome P450, *HLM* human liver microsomes, *ND* not detected, *Obsd*,observed, *Pred* predicted. ^a^Predicted to be a substrate (yes/no; percent confidence)

^b^Clearance at 1 µM expressed as µL/min/mg HLM protein. Clearance predictions for compounds predicted not to be substrates are set off by parentheses

^c^Possibly a substrate

^d^Unlikely to be a substrate

^e^Fold-errors calculated from the root mean square errors (RMSE) in the log for compounds predicted to be substrates

^f^Predicted to be an inhibitor as well as a substrate

The results for CYP2C9 and CYP2C19 in Table 3 also illustrate why it is important to make regression models for enzyme activity contingent on classification models that distinguish substrates from nonsubstrates [6, 31]. Nonsubstrates are necessarily absent from the data used to construct the regression models, and the classification models confidently identify them [32] as being out-of-scope for the corresponding regression models. The clearance predictions provided represent the values expected *if they were substrates*, which the classification models confidently predict they are not.

The situation is somewhat different for CYP1A2. Here the substrate classification predictions are uncertain—the confidences are near toss-up (41–60%), but they are still consistent with the low to nonexistent experimental clearance values for this isoform.

CYP kinetics are complicated by the potential for autoinhibition [33]. The APAQs were not tested as inhibitors, but all were predicted to inhibit CYP2D6. That may explain the consistent overestimation of CYP2D6 clearance for CYP2D6, for which the predicted affinities are high—i.e., for which the K_m_’s are all less than 1 μM (Supplementary Table S3). All four analogs are also predicted to be CYP3A4 inhibitors, but the corresponding K_m_’s are 15 μM or above, making autoinhibition unlikely to be a problem at 1 μM.

### Pharmacokinetic predictions

**Fig. 6:**
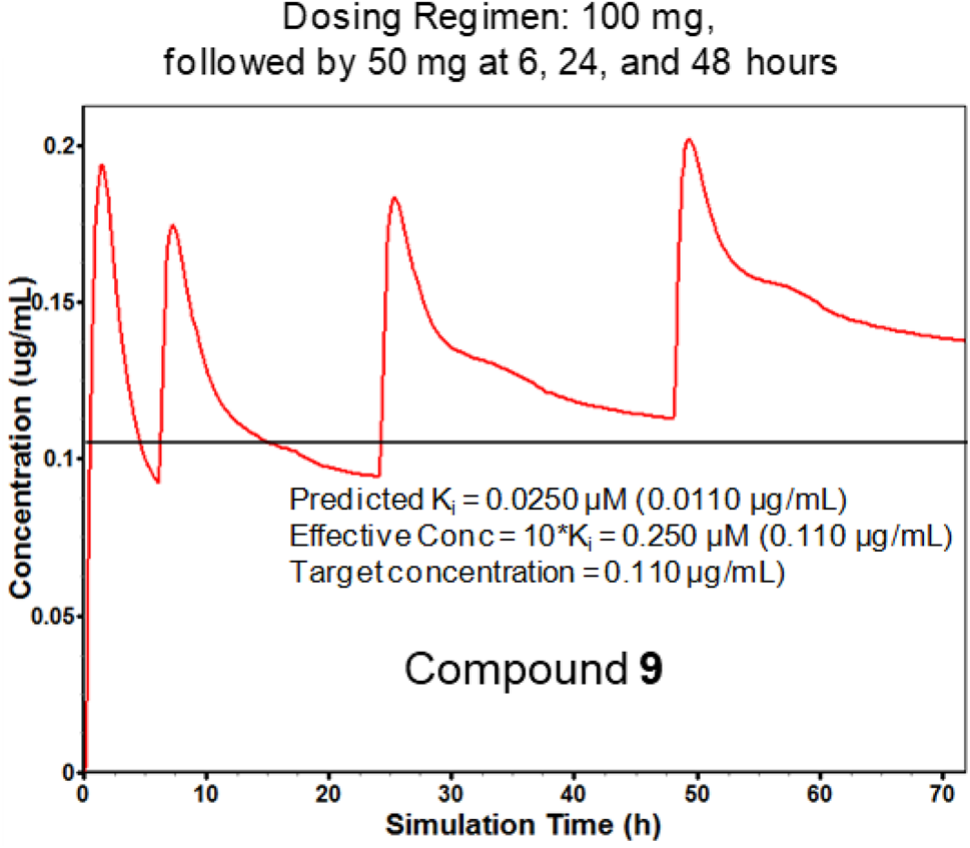
Human concentration–time profile expected for **9** based on PBPK simulation using GastroPlus. Pharmacokinetic parameters were taken from experimental values where available and estimated using the QSAR models in ADMET Predictor otherwise. * Conc* concentration,* K*_i_ predicted inhibition constant for *Pf*DHODH when plasma protein binding is taken into account

The intrinsic clearances for the most active APAQs would have posed a risk high enough to discourage further development of these compounds had they not exhibited acceptable pharmacokinetic profiles using the other property predictions. In fact, high predicted microsomal clearance was the reason several otherwise attractive candidates that failed to yield acceptable pharmacokinetic profiles were set aside during analog selection. The result for compound **9** is shown in Fig. 6. Here the predicted fraction unbound in plasma (*f*_up_ < 3%) and high volume of distribution (*V*_d_; estimated at 7 L/kg) offset the relatively high predicted intrinsic clearance. Basically, too little of the compound is likely to get into the liver for hepatic metabolism to be a problem. As a result, the bioavailability is predicted to be high enough to produce a favorable in vivo profile, given an appropriate dosing schedule—here, a schedule similar to that currently used for chloroquine.

Both f_up_ and V_d_ are in fact flagged as ADMET Risks by default, because they can make it harder for a drug to get from the plasma into peripheral tissues. That is not relevant to the blood phase of the *Plasmodium* life cycle, but would affect the ability of the compound to attack liver stages. The fact that they can offset potential first-pass metabolic liabilities is one good reason to examine pharmacokinetic simulation results for compounds of interest that raise piece-wise risk flags [31].

The QSAR predictions of *V*_d_ and *f*_up_ for **9** are similar to the values predicted for chloroquine (10.6 L/kg^2^ and 9%, respectively), which is widely used to treat malaria, and thus are not unrealistic pharmacokinetic parameters.

## Biological results

All aminoquinolones synthesized for this project were assayed for ex vivo antimalarial activity in blood culture and for inhibition of s-*Pf*DHODH. Characterization in terms of kill rate is very resource-intensive, as is determining whether or not parasites can be rescued from growth inhibition by transfection with *Saccharamyces cerevisiae* DHODH (*Sc*DHODH) DNA (see the section below on “Inhibition of intact *Pf*DHODH”). These assays were, therefore, only carried out for the two most active candidates for which sufficient material was available—**8** and **11b**. In addition, historical data for some of the APAQs in the PubChem dataset, including *Sc*DHODH rescue and ex vivo activity, were made available by the Tres Cantos Medicines Development Campus-Diseases of the Developing World, GSK (Tres Cantos) [19].

### XC_50_s for cultured parasites

Antimalarial activity in blood culture was determined as previously described [35, 36] at the University of California, Riverside, under conditions essentially identical to those used by GSK [19]. Table 4 shows the experimental results obtained in asynchronous cultures, expressed as the concentration required to reduce growth by 50% (XC_50_). With the exception of the imidazolinones **12a** and **12b**, the new compounds were more potent than the most active APAQs in the GSK data set.

**Table 4:** Antimalarial activity of APAQs in asynchronous blood culture

Compound	Pred. *Pf*DHODH	XC_50_ (µM)^a,b^		Resistance ratio (±)
	K_i_ (µM)	3d7(−)	Dd2(+)	
**12a**	0.049	10.0	46	4.6
**12b**	0.051	1.61	6.4	3.9
**11a**	0.023	0.55	2.3	4.1
**11c**	0.037	0.37	1.78	4.8
**8**	0.037	0.30	1.47	5.0
**9**	0.025	0.106	0.21	2.0
**11b**	0.038	0.037	0.24	6.6
CID 44534046^c^	0.112	0.89	4.6	5.2
CID 44535189^c^	0.077	0.85	8.6	10.1

PfDHODH dihydroorotate dehydrogenase from *Plasmodium falciparum*, *K*_*i*_ inhibition constant, *Pred* predicted

^a^Concentration required to reduce parasite growth rate by 50%

^b^(−) and (+) denote chloroquine-susceptible and -resistant strains, respectively

^c^Most active APAQs in the GSK data set

With the exception of **12a**, the novel analogs met the minimum potency threshold (better than 2 μM) for parasite proliferation called for in the Medicines for Malaria Venture (MMV) guidelines at the time [37], with **11b** satisfying the ideal criteria with an XC_50_ below 100 nM. Similar antimalarial potencies were obtained in replicate assays carried out at UC Riverside and at Tres Cantos. The compounds were also tested in the chloroquine-resistant Dd2 strain, which contains an efflux pump [38]. It was gratifying to see that the ratio of XC_50_′s between the resistant and susceptible strains—i.e., the resistance ratio (Table 4)—was lower for all of the APAQs than the value of 15.5 reported for chloroquine [39].

### Speed of killing time course assay

The parasite reduction ratio (PRR) is an indicator of how rapidly parasites are killed by antimalarial treatment [40]. Preliminary assays carried out at Tres Cantos for **8** and **11b** yielded 48-h parasite in vitro reduction ratios (PPRs) of approximately 3 log units, i.e., 99.9% of the parasites were killed in the first 48 h of exposure (1 lifecycle). This was slightly better than pyrimethamine, which was included as a positive control. Such rapid killing of the parasite is very desirable for antimalarials [41]. Little or no lag time in the onset of killing was evident for either compound, compared to the 24–48 h time lag reported for triazolopyrimidine inhibitors of *Pf*DHODH [42].

### Inhibition of s-*Pf*DHODH in vitro

Identification of a compound’s mode of action is important in drug discovery because it opens the door to structure-based drug design and because it helps alert researchers to undesirable off-target activities that might cause concern. In some cases, it highlights opportunities for synergistic inhibition of multiple targets by a single compound, i.e., for polypharmacology [43]. *Pf*DHODH was the intended target for this proof-of-principle project, so we wanted to know whether the identified lead series (in general) and our new analogs (in particular) inhibited the enzyme in intact parasites.

The literature data set used to build our *Pf*DHODH inhibition models was based on in vitro assays carried out using the truncated, soluble form of the enzyme introduced by Baldwin et al. [10]. Patel et al*.* subsequently used it to screen a library of 208,000 compounds and validated the 38 “hits” obtained by testing for inhibition of parasite growth in blood culture [8] The seven compounds we designed and synthesized were poor inhibitors of s-*Pf*DHODH with in vitro IC_50_ > 100 μM when assayed under those conditions at the Tres Cantos laboratory.

This result was somewhat unexpected, because of the active quinolones in the QSAR model training set (compounds P01 and P02 in Supplemental Table S1) and the potency of HDQ mentioned above. The substituents at the C2 position in the APAQs are longer and bulkier than are the styryl and pentyl groups in P01 and P02, but they are still much smaller than the 30- to 50-carbon polyisoprenoid tail of the natural substrate, CoQ10.

The solubility of s-*Pf*DHODH results from the removal of the hydrophobic tail that anchors the enzyme to the mitochondrial membrane, where its native cosubstrate resides in vivo. A compound that inhibits the truncated, soluble enzyme in vitro is likely to inhibit the full-length, membrane-bound form. The converse—that an inhibitor of the full-length enzyme will necessarily inhibit the truncated one—is not necessarily true, especially for inhibitors like the APAQs that resemble ubiquinone more than they resemble dihydroorotate [16]. Atovaquone, for example, inhibits the full-length enzyme from *P. falciparum* with a K_i_ of 27 µM after solubilization in Triton X-100 [44], whereas the corresponding value for the truncated enzyme is reportedly > 500 µM when assayed under similar conditions [45]. Binding to the s-*Rr*DHODH from rat is much tighter (K_i_ ~ 0.08 µM) and intermediate for the corresponding human enzyme ((K_i_ ~ 2 µM). The organization of the *N*-terminal “stump” where competitive CoQ inhibitors bind differs substantially from species to species and changes upon inhibitor binding [45]. These observations suggest that the absence of inhibition by APAQs in the in vitro assay could be due to the loss of the hydrophobic *N*-terminal residues and may not reflect the activity in living parasites.

### Inhibition of intact *Pf*DHODH

Fortunately, there is a way to address this question, though the method is somewhat indirect. *P. falciparum* parasites engineered to express DHODH from the yeast *Saccharamyces cerevisiae* (*Sc*DHODH) use fumarate as an oxidizing agent instead of ubiquinone, so their growth is not inhibited by (i.e., they are “rescued from”) inhibitors that compete with ubiquinone. Painter et al*.* used this technique to validate *Pf*DHODH as an antimalarial drug target [46].

**Fig. 7:**
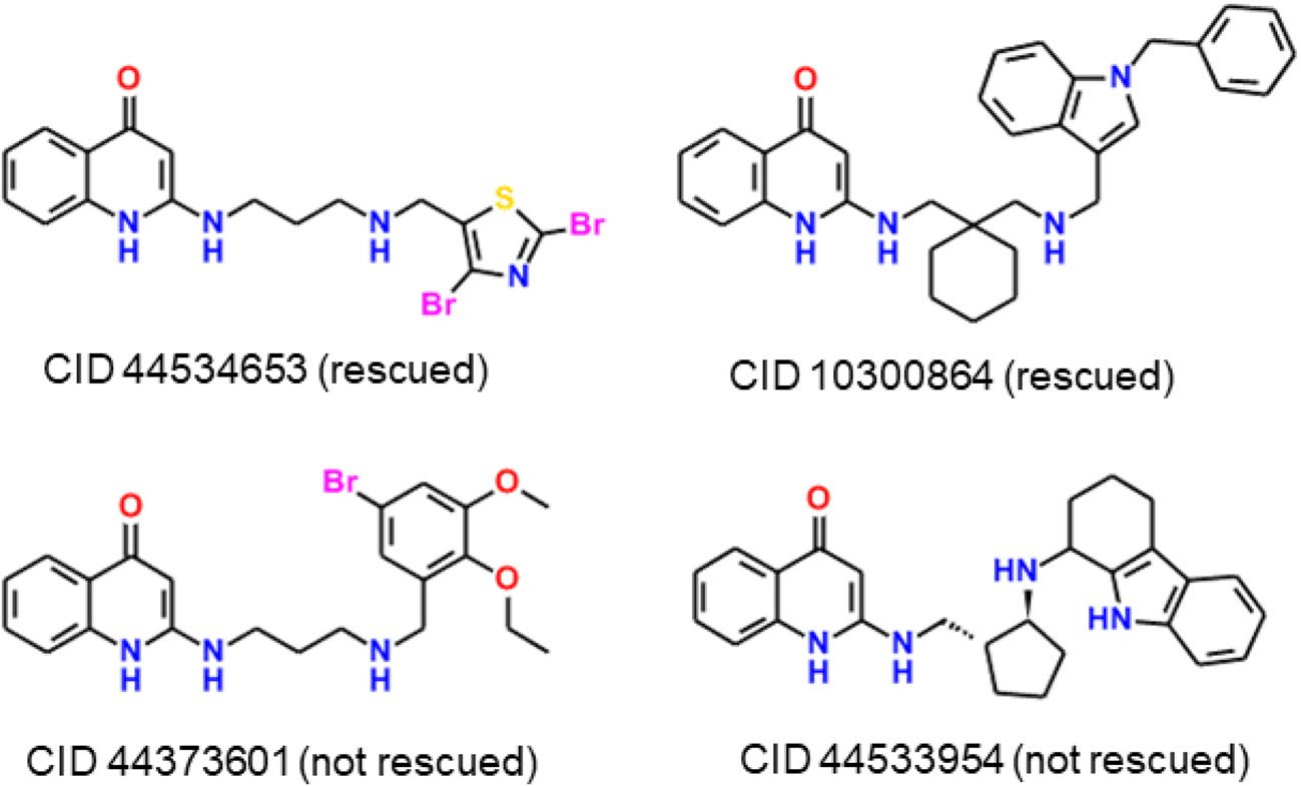
Active GSK APAQs from which *P. falciparum* grown in blood culture were rescued by transfection with *Sc*DHODH

Unbeknownst to the Simulations Plus and U.C. Riverside groups, the Tres Cantos group had already tested several 2-aminoquinolone actives from the GSK antimalarial data set for rescue by *Sc*DHODH transfection. Their previously unpublished work showed that parasites were rescued from growth inhibition by 10 of 102 APAQs assayed, with some additional compounds giving an equivocal result, i.e., partial rescue. An additional sixteen 2-amino-4-quinolones having different bridging groups were also assayed; three of these were clearly subject to rescue, whereas three yielded ambiguous results. Illustrative example structures are shown in Fig. 7.

The APAQs from which parasites were rescued by *Sc*DHODH transfection did inhibit s-*Pf*DHODH in vitro, but with higher *in vitr*o IC_50_′s (i.e., with lower affinity) than expected given the phenotypic assay results—3.1 and 5.2 μM for the most potent examples. The most potent GSK analog (CID 44,537,350) had an in vitro IC_50_ of 0.86 μM. It bore a 4-(2-pyridyl)butylamino group in place of the 3-aminopropyl amino group in our series.

The limitation of this test is that the failure of transfection to rescue parasites from the other APAQs tested—including **8** and **11b**—does not imply that those compounds do *not* inhibit the intact *Plasmodium* enzyme. It does imply, however, that *Pf*DHODH inhibition is not their *exclusive* mode of antimalarial action. Basically, **8** and **11b** start killing the parasite in culture immediately (see above), whereas specific *Pf*DHODH inhibitors take 24–48 h to do so [47]. A somewhat analogous situation exists for atovaquone, which is much more potent in blocking respiratory oxidation of ubiquinol than in inhibiting *Pf*DHODH directly [46]. In that case, the time frame and the end result—the parasite is starved of the CoQ it needs to synthesize pyrimidine nucleotides—are the same for both targets,only the target itself is different.

The bottom line is that APAQs as a class evidently do inhibit PfDHODH ex vivo but that, at least for our most potent analogs, some other, more rapid-onset mode of action is responsible for their antimalarial activity.

### Other suggested modes of action

**Fig. 8:**
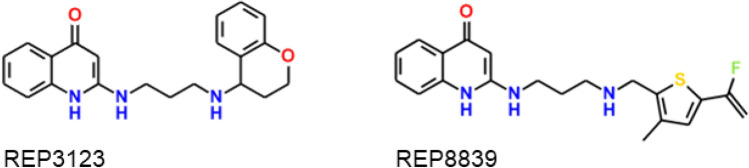
Malarial methionyl t-RNA synthetase (*Pf*MRS) inhibitors with antimalarial activity

The malarial methionyl t-RNA synthetase (*Pf*MRS) has also been suggested as a target. A patent for 2-aminoquinolones as antibiotics was filed in 1999 [27, 48] and the mode of action was characterized as being inhibition of bacterial MRS. However, the 26 compounds characterized as MRS inhibitors in that patent were not among the actives in the Gamo et al. data set [19], which argues against the idea that MRS might be the target in malaria. Subsequent to the work described here, two other APAQs characterized as bacterial MRS inhibitors (REP3123 and REP8839 in Fig. 8) were shown to inhibit the malarial enzyme in vitro and to be potent antimalarials ex vivo [49].

Those authors did not test for activity against *Pf*DHODH, however, nor did they present conclusive evidence that blocking *Pf*MRS was the compounds’ mode of action in the intact parasite*.*

In fact, the rapid knockdown and absence of a lag time in the PRR experiments argues against *any* kind of protein synthesis inhibition being involved, at least for **8** and **11b** [50].

## Conclusions

The 2-(3-aminopropylamino)-4-quinolones generated by the model-driven in silico design approach described here are potent antimalarial lead drug candidates. The methodology used to design compounds that target *Pf*DHODH yielded lead candidates with excellent activity against *P. falciparum* parasites as well as acceptable ADMET properties and favorable PK profiles. Compounds from the first design iteration were predicted to be more potent in blood culture than structurally related literature compounds and their experimentally determined potencies were consistent with that expectation. It seems likely that further iterations and in vivo characterization would be productive.

The results presented here do not pin down the primary antimalarial mode of action for the APAQs, but do indicate that it is probably neither *Pf*DHODH nor protein synthesis. Fortunately, the lack of cytotoxicity the lead series showed against mammalian cells in culture suggests that inhibition of the primary target, whatever it is, is not intrinsically problematic with respect to human beings. If that is indeed the case, having multiple activities should improve prospects for further development iterations centered around this class of chemistry, since hitting multiple targets is a good way to slow development of resistance.

This successful proof-of-principle study highlights how useful in silico tools can be in de novo design and for identifying lead compounds from phenotypic screens. The example chosen illustrates how including predictive PBPK simulations early in the workflow can reduce the risk of relying too heavily on any single ADMET property filter when selecting analogs for synthesis. Applied more generally, the overall approach has the potential to reduce the number of compounds that need to be synthesized to get from discovery through optimization, to make animal testing more efficient, and to reduce attrition in clinical trials. Beyond being useful as a proof-of-principle, the exercise provided prospective validation of several kinds of in silico property predictions as well as an example of the kind of unexpected off-target activity commonly encountered in drug development projects—in this case, a desirable one.

## Electronic supplementary material

Below is the link to the electronic supplementary material.

Supplementary file1 (XLSX 362 kb)—Supplemental Information: Data.Supplementary Table S1 contains structures and Ki values used to build the PfDHODH inhibition models. Structuresof the original 12 synthesis candidates are provided in Supplementary Table S2, along with their predictedPfDHODH Ki’s and critical ADME property predictions. Data on the four analogs that were synthesized and fullycharacterized (8, 9, 11a and 11b) are given in Supplementary Tables S3 and S4. Supplementary Table S3 comparesmetabolism predictions from ADMET Predictor v9.0 with those from v6.0 and with the corresponding experimental values. Predictions of the corresponding predicted and experimentally determined physicochemical properties areprovided in Table S4.

Supplementary file2 (DOCX 207 kb)—Supplemental Information: MethodsMethodological details concerning computations, assays, synthesis and chemical characterization of products areprovided in the supporting materials.

## Data Availability

Our supply of synthesized material is exhausted at present, but the experimental procedures required to synthesize more are provided. NMR spectra and any details not included in the supplementary materials are available from the authors upon request.

## Footnotes

1 A prediction was flagged as “out of scope” when any of its descriptor values fell more than 10% outside the range of the corresponding values seen for the data set upon which the model was trained.

2 The actual V_d_ for chloroquine is difficult to determine experimentally, but is probably much higher [34].

## Code availability

ADMET Predictor® and GastroPlus® are commercially available from Simulations Plus, Inc., Lancaster CA 93534 (https://www.simulations-plus.com).
