# Supplementary material for: Design and tests of prospective property predictions for novel antimalarial 2-aminopropylaminoquinolones
Source: J Comput Aided Mol Des. 2020 Aug 24;34(11):1117–32. doi: 10.1007/s10822-020-00333-x (PMC7533260; doi:10.1007/s10822-020-00333-x)
Supplement: Supplementary file 2 — Supplementary file2 (DOCX 207 kb)—Supplemental Information: MethodsMethodological details concerning computations, assays, synthesis and chemical characterization of products areprovided in the supporting materials. [file 10822_2020_333_MOESM2_ESM.docx]

**Design and tests of prospective property predictions for novel antimalarial**

**2-­aminopropylaminoquinolones: Supplementary Methods**

Robert D. Clark^1*^, Denise N. Morris^2^, Gary Chinigo^3‡^, Michael S. Lawless^1^, Jacques Prudhomme^4^, Karine G. Le Roch^4,a^, Maria José Lafuente^5^, Santiago Ferrer^5,b^, Francisco Javier Gamo^5^, Robert Gadwood^3^, Walter S. Woltosz^1^

^1^Simulations Plus, Inc., Lancaster, CA, USA

^2^Cognigen Corporation, a Simulations Plus Company, Buffalo, NY, USA

^3^Kalexsyn, Inc., Kalamazoo, MI, USA

^4^Department of Molecular, Cell and Systems Biology, University of California, Riverside, CA, USA

^5^Tres Cantos Medicines Development Campus-Diseases of the Developing World, GlaxoSmithKline, Tres Cantos, Madrid, Spain

*Correspondence:

Robert D Clark

Sr. Research Fellow

Simulations Plus, Inc.

42505 10th Street West, Lancaster, CA 93534-7059

Phone: +1 661 723 7723

Fax: +1 661 723 5524

[bob@simulations-plus.com](mailto:bob@simulations-plus.com)
[ORCID: 0000-0001-9509-8132](https://orcid.org/0000-0001-9509-8132)

^‡^ Current affiliation: Pfizer Inc., Groton, CT, USA

^a^ ORCID: 0000-0002-4862-9292
^b^ ORCID: 0000-0002-6672-551X

Key Words: antimalarial; ADME; DHODH; dihydroorotate dehydrogenase; drug design; PBPK; QSAR; QSPR; aminopropylaminoquinolone.

### **Computational Methods**

### **QSAR modeling and predictions**

*K*_i_ and property predictions were generated by artificial neural net ensemble (ANNE) models built using the ADMET Modeler™ Module in ADMET Predictor 6.0 [1]. The activity models were built on log-transformed endpoints. Hence their predictive performance - which was assessed against a held-out test set (>10%) – is given as RMSE in log units or as a fold-error (10^RMSE^) for endpoints like solubility and clearance, which were back-transformed to a linear scale.

Our general approach for building QSAR and QSPR models has been described in detail elsewhere [2]. For the particular case of the *Pf*DHODH inhibition models, the number of inputs available for descriptor selection n ADMET Modeler™ was reduced by excluding simple elemental counts and substructure counts (e.g., the number of carbon atoms or number of sulfonamide groups). Sixty-four (64) candidate descriptors remained after low-variance, underrepresented and inter-correlated ones were removed. Test sets were created by stratified sampling, taking every fifth compound from a sorted list starting at the 3^rd^ or 4^th^ lowest *K*_i_.

Several different random number seeds were examined for initializing artificial neural network weights and for partitioning observations in the training pool between training and verification sets across the 33 networks in each ensemble. In addition, several different architecture grids (ranges of neuron and descriptor counts as well as intervals for each) were examined. Such perturbations were explored to help ensure that the models obtained were robust and representative in terms of their performance. Any prediction for a compound whose descriptors fell more than 10% outside the range of model descriptors seen in the data set used to train that model was classified as being “out of scope” [2]. APAQs exist as an equilibrium mixture of hydroxyquinoline and quinolone tautomers, with ^1^NMR spectra indicating that the quinolone tautomer dominates in most cases. Predicted inhibition and property values were therefore based primarily on the quinolone forms. The exception was pK_a_ prediction, where the hydroxyquinoline tautomers were used. This was necessary because, like most pK_a_ prediction programs, ADMET Predictor does not currently recognize ketones as protonatable centers.

Version 6.0, which was released in 2011, was employed for the *Pf*DHODH model building and for the original virtual library screening, whereas version 9.0 was used to compare predictions with experimental results. ADMET properties were consistently better in the more recent version but the differences in accuracy were small for physicochemical properties (Supplementary Table S4). Those changes that are seen reflect changes in the data sets used to train the models that produced them; enhancements in pK_a_ predictions are a case in point [3].

No model for overall microsomal intrinsic clearance was available during the design phase of the project. Moreover, the individual CYP clearance predictions were mostly flagged as being “out of scope,” i.e., as being too dissimilar to the training set for predictions to be reliable (Supplementary Table S3). Those predictions were therefore given little weight when selecting analogs for synthesis.

Rebuilding the CYP models on expanded data sets has improved the accuracy and broadened the scope of the metabolism models in ADMET Predictor, with the result that APAQ intrinsic clearances are now in-scope and reasonably well-predicted. Those predictions are also still unbiased, in that the data for them have never been included in ADMET Predictor’s training sets.

Applicability domains are defined by the range of descriptor values in the training set plus or minus 10% of the range of each value. rCYP clearance predictions were corrected for partial saturation by applying the Michaelis-Menten affinities (K_m_’s) predicted by ADMET Predictor 9.0.

### **Lead series identification**

MedChem Studio was used to generate structural classes from the GSK actives. The algorithm used identifies core shared substructural “anchors”, which are elaborated into the distinctive maximal common substructures that define each class. Because the classes generated are based on shared substructures rather than substructural fingerprints, the overlapping classes produced represent chemical series [4,5].

Class generation was set to start from rings and ring systems augmented by any nonaromatic oxygen and nitrogen substituents. The homogeneity and redundancy parameters control the minimum size of the scaffold with respect to the largest member of a class and the degree to which class memberships are allowed to overlap, respectively; both were set to “medium.” Of the 1061 classes identified, the 90 doubletons and 283 singletons were set aside. The class member with the lowest predicted *Pf*DHODH K_i_ (Model A) was chosen as the representative for each class, after which classes having the same representative were combined. A new scaffold was defined for the combined classes either by finding the maximum common substructure or manually in such a way that the new scaffold was chemically meaningful and “hit” most compounds in both of the original classes.

The default ADMET Risk rules from ADMET Predictor 6.0 were used without modification. These 24 rules taken into consideration addressed 24 calculated or predicted properties: size, number of freely rotatable bonds, number and strength of hydrogen bond donors; number and strength of hydrogen bond acceptors; overall molecular charge; hydrophobicity; effective permeability; water solubility; fraction unbound in plasma; volume of distribution; CYP metabolism and inhibition (seven rules); hERG binding; acute toxicity to rats; chronic toxicity to rats or to mice (two rules); Ames mutagenicity; and hepatotoxicity (two rules). “Undecided” *in silico* Ames predictions contributed 0.5 to the ADMET Risk score; otherwise all rules were given equal weight.

MedChem Studio evolved from ChemTK from Sage Informatics and the ClassPharmer program developed by Bioreason and became a module of ADMET Predictor 8.0. MedChem Studio 1.0 was used for the analysis described here, but the illustrative plots shown are from the MedChem Studio Module in ADMET Predictor 9.0.

### **Virtual library enumeration and analog vetting**

The 2-(3-aminopropylamino)quinolone scaffold from the GSK actives included examples with substituents at the 3, 5, 6, and 7 positions on the quinolone ring as well as at the 2’, 3’, and distal amine (*N*”) of the aminopropylamino bridge. High lipophilicity was a problem for the class as a whole, so the combinatorial design was restricted to keep quinolone and bridge substituents small. Most of the complexity was confined to the distal nitrogen substituent, and only analogs with a single substituent at that nitrogen were considered.

A virtual library was produced using the R-Table Explosion option in MedChem Studio. Analogs remaining after screening out compounds predicted to have low activity or high ADMET Risk were split into four classes in MedChem Studio using a homogeneity setting of “high” and a redundancy setting of “low.”

An initial set of synthesis candidates was manually selected from among the structures in each class that combined low predicted *K*_i_ with low predicted ADMET Risk – i.e., the safest, most active compounds. Those from Class 3 bore a *gem*-cyclohexyl ring in the middle of the central aminopropylamino group, whereas those from Class 4 bore a vicinal 1,2-cyclopentyl ring, so the possibility of replacing it with a gem-cyclopentyl or -tetrahydrofuranyl ring was examined as a way to increase solubility while retaining activity; the cyclopentyl variant was likely to be simpler from a synthetic standpoint and was predicted to be more active. Neither was represented among the GSK actives but both were predicted to be more active and to have lower ADMET Risk scores than similar GSK actives.

### **PBPK simulations**

GastroPlus [6] combines species-specific parameters (e.g., blood flow rates, organ sizes, and composition) with compound-specific ADMET properties (e.g., pK_a_, solubility, and metabolic lability) to set up a series of differential equations based upon intercompartmental flow and mass balances. These can be solved numerically to simulate the plasma and tissue concentration-versus-time profiles of a compound in the animal or human system of interest. The compound-specific properties used in such simulations can be predicted *in silico* or drawn from experimental results. The former source of inputs is useful when predicting pharmacokinetic properties prior to synthesis. This was done using only physicochemical properties predicted using ADMET Predictor 6.0 for fully prospective PBPK simulations used to design the analogs described herein. Microsomal clearance was estimated as the sum of predicted intrinsic clearance contributions from CYPs 1A2, 2C9, 2C19, 2D6 and 3A4, because no model for total CYP HLM clearance was available at the time. Simulated profiles for two APAQs that were not synthesized – one (**13**) where the target systemic concentration was not expected to be sustained and another (**4**) where it was – are shown in Fig. 9.


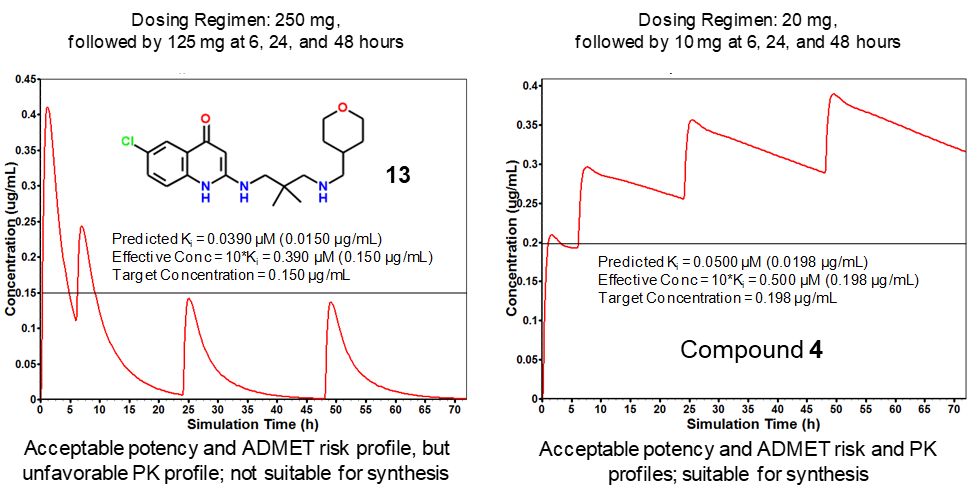


**Fig. S1:** Projected pharmacokinetic (PK) profiles for lead candidates generated in GastroPlus using the QSAR-based estimates from ADMET Predictor. The profile on the left is unacceptable, whereas that on the right is acceptable. The dosing regimen used is similar to that used for chloroquine. Conc, concentration; K_i_, *in vitr*o inhibition constant predicted for *Pf*DHODH.

This is in contrast to the simulation shown for **9** in Fig. 6 in the main text, for which experimental values for physicochemical properties and CYP clearance in human liver microsomes were used.

### **Chemistry**

Unless otherwise stated, all externally sourced chemicals were reagent grade or better and were obtained from Sigma-Aldrich.

Analytical HPLC was carried out on an Agilent 1100 HPLC. Zorbax Eclipse XDBC18 50 x 4.6 mm 1.8 micron column. Solvent A was water-containing and Solvent B was acetonitrile, containing 0.1 and 0.07% TFA, respectively. Sample was eluted by applying a 95%A to 95%B gradient over a 5-6 min interval. UV detection was at 254 and 210 or 214 nm.

Internal identifiers for final products (e.g., “SLP0001”) used in preliminary communications are included in the experimental details for reference.

Yields were not optimized. Several products isolated as oils or foams that subsequently solidified into glassy solids.

**Structures**

For the sake of simplicity, structure numbering follows that used in the main article. Structures are shown in Fig.S2.


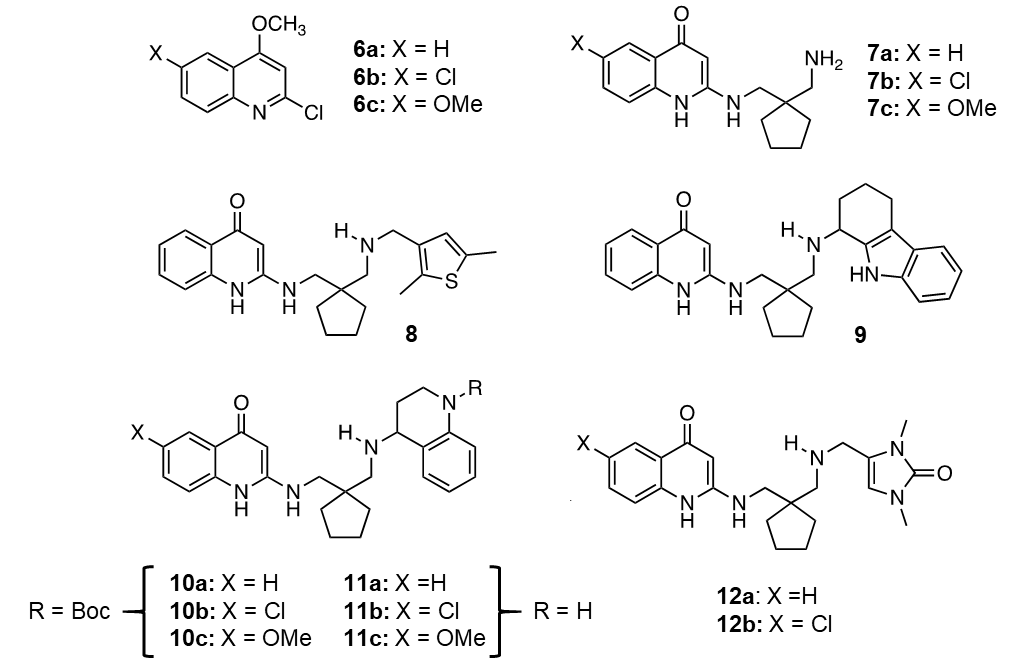


Fig. S2: Structures of main intermediates and products. Compound labels are taken from the main article.

### **1-(Aminomethyl)cyclopentylmethanamine** (**5**)

Sodium hydride (21.0 g, 0.53 mol) was added at 0°C to a mixture of 15.8 g malononitrile (0.24 mol) and 34 mL 1,4-dibromobutane (0.29 mol) in 400 mL *N,N*-dimethylformamide (DMF). The mixture was allowed to warm to room temperature and stirred for 3 hr, then the reaction was quenched with water and extracted with hexanes and methyl t-butyl ether (MTBE). The combined organic layers were pooled, dried with MgSO_4_, and concentrated under vacuum to give 65 g of crude 1,1-dicyanocyclopentane (~100% yield) that included 1 equivalent of DMF and 0.2 equivalent of dibromobutane.

LiAlH_4_ (7.2 g, 0.19 mol) was added slowly to 210 mL diethyl ether held at 0°C. A solution of 7.0 g (58 mmol) of 1,1-dicyanocyclopentane dissolved in 50 mL diethyl ether was added dropwise, slowly enough to keep the reaction mixture’s temperature below 10°C. The suspension was stirred overnight at room temperature, then cooled to 0°C and quenched with 7.2 mL water, followed by 7.2 mL 15% NaOH, then with 21.6 mL water. The suspension was filtered through Celite and the filter cake washed with tetrahydrofuran. The filtrate was concentrated under vacuum to give 6.05 g 1-(aminomethyl)cyclopentylmethanamine, a yield of 81%. The product obtained was used without further purification.

### **2-Chloro-4-methoxyquinolines** (**6a-c**)

4-Methoxy-1H-2-quinolone was prepared from 4-hydroxy-1H-2-quinolone and converted to 2-chloro-4-methoxyquinoline (**6a**) as described by Chen *et al*. [7], except that trimethylamine was substituted for trimethylamine. Yield was 85%.

2,4-Dichloro-6-methoxyquinoline was prepared as described by Yang *et al.* [8] and converted to 2-chloro-4,6-dimethoxyquinoline (**6c**) as described by Okubo *et al*. [9] in 57% yield.

### **2,6-Dichloro-4-methoxyquinoline**

A solution of 70.0 g methyl 2-amino-5-chlorobenzoate (AK Scientific; 0.38 mol) in 527 mL N-methylpyrrolidinone was stirred in a 2L 3-necked round-bottom flask equipped with an addition funnel and cooled to 0°C. After 39.0 mL chloroacetyl chloride (0.49 mol) was added dropwise over 30 min, the cooling bath was removed and the mixture was allowed to warm to room temperature. The mixture was then diluted by addition of 800 mL water, which resulted in an exothermic reaction. After cooling to room temperature, the solid was collected by filtration and washed with water. Drying overnight at 60°C yielded 97.8 g of product with a ^1^H NMR consistent methyl 5-chloro-2-(chloroacetyl)aminobenzoate. The intermediate was >95% pure by HPLC. Yield was ~99%.

A mixture of 65 g 5-chloro-2-(chloroacetyl)aminobenzoate (0.25 mol) and 72 g triphenylphosphine (0.27 mol) in 320 mL DMF was heated at 80°C. A yellowish-white precipitate formed after 1 hr; after 17.5 hr, the reaction mixture was cooled to room temperature with 500 mL 1:1 ethyl acetate:MTBE (EA/MTBE). The precipitate produced was collected by filtration and washed with an additional 500 mL EA/MTBE to yield 85 g of a white solid, which was taken up in 800 mL 1:1 chloroform:water and neutralized by addition of 1M NaOH until all solids had dissolved; the pH was ~11 at that point. The aqueous phase was drawn off and re-extracted twice with 150 mL chloroform. The combined organic phases were dried over MgSO_4_, then concentrated under vacuum overnight.

The 77 g of methyl 5-chloro-2-(triphenylphosphoranylideneacetyl)aminobenzoate produced was placed in a round bottom flask that was evacuated and flushed with nitrogen three times, then heated at 180°C for 1.5 hr. After cooling to room temperature, 500 mL 1:1 ethyl acetate/diethyl ether was added and the slurry stirred with gentle heating. Solids were scraped from the sides of the flask. Solids were filtered off and triturated twice with 200 mL EA/MTBE to remove triphenylphosphine oxide. Trituration with chloroform (once with 200 mL and once with 150 mL) gave a light yellow solid that was dried overnight under high vacuum to yield 21.0 g (~63%) of product. Purity by HPLC was >95% and the ^1^H NMR was consistent with that expected for 6-chloro-4-methoxy-1*H*-2-quinoline, which was converted to the desired 2,6-dichloro-4-methoxyquinoline (**6b**) as described above for the 6-H analog **6a**.

### **Aminopropylaminoquinolones**

### 2-(1-(aminomethyl)cyclopentyl)methylamino-1H-quinolin-4-one (**7a**; SLP0001)

A mixture of 2.1 g **5** (16 mmol), 2.44 g **6a** (12.6 mmol), 0.601 g dicyclohexyl(2’,4’,6’-triisopropylbiphenyl-2-yl)phosphine (XPhos; 1.26 mmol), 2.42 g tris(ddibenzylideneacetone)dipalladium(0) chloroform adduct (Pd2(dba3·CHCl_3_); 1.26 mmol) and 1.30 g sodium t-butoxide (25.2 mmol) were microwaved for 10 min at 112°C in 15 mL 1,4-dioxane. The reaction mixture was concentrated under vacuum and purified by flash chromatography in 12.5:1 DCM:7 N NH_3_ in methanol (DCM/ NH_3_/MeOH).

A portion (0.55 g, 1.9 mmol) of the 2.0 g of 4-methoxy intermediate obtained (56% yield) was hydrolyzed by refluxing overnight in 30 mL 5N aqueous HCl. The cooled mixture was neutralized with ammonium hydroxide, and the aqueous layer extracted repeatedly with 5:1 DCM/isopropyl alcohol and the combined organic layers dried over MgSO_4_. Flash chromatography using DCM/ NH_3_/MeOH yielded the 0.44 g of the desired **7a** as an off-white foam. The material was >95% pure by HPLC with UV detection, but 0.73 mol:mol residual isopropyl alcohol was evident by NMR;, yield 84%.

^1^H NMR (400 MHz, CD_3_OD) δ (ppm): 8.15 (br d, J=8.0 Hz, 1H), 7.90 (br d, J=9.2 Hz, 1H), 7.80 (td, J=7.8, 1.6 Hz, 1H), 7.51 (br t, J=8.0 Hz, 1H), 6.56 (s, 1H), 3.60 (s, 2H), 3.18 (s, 2H), 1.82 (br m, 4H), 1.75 (br m, 4H); ESI-MS (*m/z*): 272.3 [M+H]^+^, 270.3 [M-H]^-^.

### 2-(1-(aminomethyl)cyclopentyl)methylamino-6-chloro-1H-quinolin-4-one (**7b**; SLP0002).

Prepared from **6b** as described above for the 6-H analog **7a**. Colorless foam >95% pure by HPLC; ^1^H NMR (400 MHz, CD_3_OD) δ (ppm): 8.03 (d, J=2.4 Hz, 1H), 7.41 (dd, J=8.6, 2.4 Hz, 1H), 7.37 (d, J=8.8 Hz, 1H), 5.70 (s, 1H), 3.27 (s, 2H), 2.66 (s, 2H), 1.71 (br m, 4H), 1.59 (br m, 2H), 1.47 (br m, 2H); ESI-MS (*m/z*): 306.2 [M+H]^+^, 304.3 [M-H]^-^.

### 2-(1-(aminomethyl)cyclopentyl)methylamino-6-methoxy-1H-quinolin-4-one (**7c**; SLP0009)

Prepared from **6c** as described above for the 6-H analog **7a**. Colorless foam, >99% pure by HPLC; ^1^H NMR (400 MHz, CD_3_OD) δ (ppm): 7.56 (d, J=2.8 Hz, 1H), 7.30 (d, J=8.8, 1H), 7.10 (dd, J=8.8 Hz, 3.2, 1H), 5.69 (s, 1H), 3.84 (s, 3H), 3.19 (s, 2H), 2.61 (s, 2H), 1.67 (br m, 4H), 1.53 (br m, 2H), 1.42 (br m, 2H); ESI-MS (*m/z*): 302.2 [M+H]^+^, 285.2 [M-NH_3_+H]+, 300.3 [M-H]^-^.

### Reductive N-alkylation with ketones

### 2-(1-((2,3,4,9-tetrahydro-1H-carbazol-1-yl)aminomethyl)cyclopentyl)methylamino-1H-quinolin-4-one *(****9;*** *SLP0003).*

Aminopropylamino quinolone **7a** (0.36 g, 1.3 mmol), 0.24 g 2,3,4,9-tetrahaydro-1H-carbazole-1-one (1.3 mmol), 0.612 g MgSO4 (5.1 mmol), 0.612 g molecular sieves (5.14 mmol), and 0.28 mL acetic acid (5.0 mmol) were taken up in 20 mL ethanol. After stirring at 65°C for 24 hr, 0.167 g sodium cyanoborohydride (2.65 mmol) was added and reaction continued for 12 hr at 60°C. Solvent was then removed under vacuum and the residual material was partitioned between water and DCM. The organic layer was drawn off and the aqueous phase was re-extracted with additional DCM. The combined organic layers were dried with MgSO_4_ and concentrated under vacuum. Repeated CombiFlash® Rf chromatography using a 0 to 7N methanolic NH_3_ gradient in DCM was followed by trituration with methanol to yield an off-white foam that was >95% pure by HPLC. The chromatographic peak was broad, even when run at 45°C. The ^1^H NMR peaks were also broad but did sharpen at higher temperature. That suggests slow exchange between multiple conformers rather than impurity, which is consistent with the chromatographic behavior.

^1^H NMR (400 MHz, DMSO-*d*_6_) δ (ppm): 11.16 (br 1H), 11.10 (br 1H), 7.89 (dm, J=8.0 Hz, 1H), 7.38 (dd, J=9.0, 9.0 Hz, 2H), 7.17 (m, 1H), 7.06 (m, 2H), 6.96 (dd, J=7.2, 7.2 Hz, 1H), 6.85 (br d, J=8.4 Hz, 1H), 6.65 (br, 1H; NH?), 5.58 (s, 1H), 3.91 (br, 1H), 3.22 (m, 1H), 3.13 (m, 1H), 2.66 (d, J=12 Hz, 1H), 1.91 (br, 2H), 1.62 (br m, 8H), 1.51 (br m, 4H); ESI-MS (*m/z*): 441.4 [M+H]^+^, 439.3 [M-H]^-^.

### 2-(1-((1,2,3,4-tetrahydroquinolin-4-yl)aminomethyl)cyclopentyl)methylamino-1H-quinolin-4-one (**11a**; SLP0004).

The *N*-protected intermediate **10a** was prepared from *N*-t-butoxycarbonyl-3,4-dihydro-4-quinolone and **7a** as described above for the tetrahydrocarbazole analog **9**. The Boc protecting group was removed by transamidation and subsequent base hydrolysis: 0.13 g **10a** (0.26 mmol) was stirred in 2mL formic acid at room temperature for 16 hr, at which point the solvent was removed under vacuum and the residue refluxed in 4N NaOH for 20 hrs. Once the reaction mixture had cooled to room temperature, it was partitioned between water and 4:1 DCM/isopropanol. The organic layer was dried with MgSO_4_ and then concentrated under vacuum. The residue was purified by flash chromatography using 9:1 DCM/NH_3_/MeOH, followed by CombiFlash® Rf chromatography to yield afford a colorless foam that was >97% pure, balance isopropanol. The yield was 48%.

^1^H NMR (400 MHz, CD_3_OD) δ (ppm): 8.08 (dd, J=8.2, 1.2 Hz, 1H), 7.44 (ddd, J=7.9, 7.5, 1.4 Hz, 2H), 7.23 (m, 1H), 7.16 (d, J=8.4 Hz, 1H), 7.13 (d, J=7.6 Hz, 1H), 6.97 (ddd, J=8.4, 7.6, 1.4 Hz, 1H), 6.57 (ddd, J=8.3, 7.7, 1.4 Hz, 1H), 6.54 (dd, J=8, 0.8 Hz, 1H), 5.68 (s, 1H), 3.88 (t, J=5.0 Hz, 1H), 3.27 (m, 1H), 3.25 (m, 1H), 3.23 (m, 1H), 2.74 (d, J=12.0 Hz, 1H), 2.68 (d, J=12 Hz, 1H), 1.90 (m, 1H), 1.84 (m, 1H), 1.70 (m, 4H), 1.45-1.65 (m, 4H); ESI-MS (*m/z*): 403.4 [M+H]^+^, 401.3 [M-H]^-^.

### 6-chloro-2-(1-((1,2,3,4-tetrahydroquinolin-4-yl)aminomethyl)cyclopentyl)methylamino-1H-quinolin-4-one (**11b**; SLP0006)

Prepared from **6b** as described above for the 6-H analog **11a**. Colorless foam; >95% pure by HPLC; ^1^H NMR (400 MHz, CD_3_OD) δ (ppm): 8.01 (d, J=3.2 Hz, 1H), 7.35 (dd, J=11.6, 3.2 Hz, 1H), 7.13 (d, J=10.0 Hz, 1H), 7.04 (d, J=11.6 Hz, 1H), 6.98 (ddd, J=10.1, 9.0, 8, 1.4 Hz, 1H), 6.58 (ddd, J=10.0, 9.2, 1.2 Hz,1H), 6.54 (d, J=9.6 Hz, 1H), 5.66 (s, 1H), 3.87 (t, J=6.8 Hz, 1H), 3.26 (m, 2H), 3.22 (m, 1H), 3.17 (m, 1H), 2.74 (d, J=15.6 Hz, 1H), 2.65 (d, J=15.6 Hz, 1H), 1.86 (br m, 2H), 1.70 (m, 4H), 1.45-1.65 (m, 4H); ESI-MS (*m/z*): 437.3 [M+H]^+^, 435.4 [M-H]^-^.

### 6-methoxy-2-(1-((1,2,3,4-tetrahydroquinolin-4-yl)aminomethyl)cyclopentyl)methylamino-1H-quinolin-4-one (**11c**; SLP0010)

The *N*-protected intermediate **10c** was prepared as described above for the 6-H analog **10a**. To remove the Boc protecting group, 0.40 g 10c (0.74 mmol) and 1.01 g ZnBr_2_ (4.47 mmol) were mixed in 6 mL DCM at room temperature with periodic sonication. Two additional 1.01 g boluses of ZnBr_2_ were at 3 hr intervals, with 5 min sonication after each addition. After stirring overnight at room temperature, the white suspension was diluted with water and 4:1 DCM/isopropanol. The organic layer was drawn off and dried with MgSO4 and solvent removed under vacuum. CombiFlash® Rf chromatography in 9:1 DCM/NH_3_/MeOH yielded the desired product as 0.101 g of a colorless foam that was >99% pure by HPLC. Yield was 31%.

^1^H NMR (400 MHz, CD_3_OD) δ (ppm): 7.56 (s, 1H), 7.14 (d, J=10.0 Hz, 1H), 7.06 (s, 2H), 6.98 (dd, J=10.8, 9.2 Hz, 1H), 6.59 (d, J=10.0 Hz,1H), 6.55 (d, J=10.4 Hz, 1H), 5.68 (s, 1H), 3.89 (t, J=3.0 Hz, 1H), 3.86 (s, OMe 3H), 3.28 (d, J=5.6 Hz, 2H), 3.25 (m, 1H), 3.18 (m, 1H), 2.76 (d, J=15.6 Hz, 1H), 2.67 (d, J=15.6 Hz, 1H), 1.80 (m, 2H), 1.72 (m, 4H), 1.56 (m, 4H); ESI-MS (*m/z*): 433.2 [M+H]^+^, 302.2 [M-dihydroquinoline]^+^, 431.3 [M-H]^-^.

### **Reductive N-alkylation with aldehydes**

### 4.2.5.1. 2-(1-((2,5-dimethyl-3-thienyl)methylaminomethyl)cyclopentyl)methylamino-1H-quinolin-4-one (**8**; SLP0005)

Compound 7a (0.170 g, 0.626 mmol) and 0.0878 g 2,5-dimethylthiophene-3-carbaldehyde (0.626 mmol) were dissolved in 4 mL and 0.0787 g sodium cyanoborohydride was added. The reaction ceased progressing after 1.5 hr, so one additional equivalent each of cyanoborohydride and of aldehyde were added. After 3 more hours, the reaction mixture was concentrated under vacuum and purified by flash followed by CombiFlash® Rf chromatography using DCM/NH_3_/MeOH. Elution gradients of 0 to 3 to 6% and 0 to 8%, respectively, yielded 0.132 g of the desired product as a colorless solid that was >98% pure; the balance was DCM.

^1^H NMR (400 MHz, CD_3_OD) δ (ppm): 7.95 (d, J=8.4 Hz, 1H), 7.37 (ddd, J=8.2, 7.6, 1.2 Hz, 1H), 7.12 (dd, J=8.0, 7.6 Hz, 1H), 7.11 (d, J=8.0 Hz, 1H), 6.47 (s, 1H), 5.66 (s, 1H), 3.65 (s, 2H), 3.18 (s, 2H), 2.48 (s, 2H), 2.19 (s, 3H), 2.11 (s, 3H), 1.55 (br m, 6H), 1.33 (br m, 2H); ESI-MS (*m/z*): 396.5 [M+H]^+^, 394.3 [M-H]^-^.

### 2-(1-((1,3-dimethyl-2-oxo-imidazol-4-yl)methylaminomethyl)cyclopentyl)methylamino-1H-quinolin-4-one (**12a**; SLP0007)

Prepared from **7a** and 1,3*-*dimethyl-1,3-dihydro-2H-imidazol-2-one as described above for the thienylmethyl analog **8** in a yield of 41%. Colorless foam; >97% pure, balance isopropyl alcohol; ^1^H NMR (400 MHz, CD_3_OD) δ (ppm): 8.09 (dd, J=10.8, 1.6 Hz, 1H), 7.53 (ddd, J=10.2, 9.4, 2.0 Hz, 1H), 7.34 (d, J=10.8, 1H), 7.26 (ddd, J=10.5, 10.2, 1.2 Hz, 1H), 6.39 (s, 1H), 5.73 (s, 1H), 3.66 (s, 2H), 3.29 (s, 2H), 3.24 (s, 3H), 3.21 (s, 3H), 2.56 (s, 2H), 1.60-1.75 (br m, 4H), 1.59 (br m, 2H), 1.47 (br m, 2H); ESI-MS (*m/z*): 396.2 [M+H]^+^, 394.3 [M-H]^-^.

### 6-chloro-2-(1-((1,3-dimethyl-2-oxo-imidazol-4-yl)methylaminomethyl)cyclopentyl)methylamino-1H-quinolin-4-one (**12b**; SLP0008)

Prepared from **7b** as described above for the 6-H analog **12a** in a yield of 45%. Colorless foam; >95% pure, trace of free amino precursor; ^1^H NMR (400 MHz, CD_3_OD) δ (ppm): 8.03 (d, J=3.2 Hz, 1H), 7.47 (dd, J=11.6, 3.2 Hz, 1H), 7.29 (d, J=11.6, 1H), 6.38 (s, 1H), 5.68 (s, 1H), 3.63 (s, 2H), 3.26 (s, 2H), 3.24 (s, 3H), 3.21 (s, 3H), 2.52 (s, 2H), 1.60-1.75 (br m, 4H), 1.57 (br m, 2H), 1.47 (br m, 2H); ESI-MS (*m/z*): 430.2 [M+H]^+^, 428.3 [M-H]^-^.

### **Ancillary reagents**

### N-t-Butoxycarbonyl-3,4-dihydro-4-quinolone

Di-t-butyl dicarbonate (Boc_2_CO; 0.642 g, 2.9 mmol) was added to a suspension of 0.45 g 2,3- dihydro-4-quinolone hydrochloride (2.4 mmol) and 0.449 g 4-dimethylaminopyridine (DMAP; 3.7 mmol) in 20 mL acetonitrile. Reaction was incomplete after stirring overnight at 50°C, so a total of 0.40 g more Boc_2_CO (2.3 mmol) was added over the next hour. Reaction was complete after two more hours at 50°C, at which point volatiles were removed under vacuum. The residue was purified by flash chromatography in 4:1 ethyl acetate/hexanes to yield 0.71 g *N*-t-butoxycarbonyl-3,4-dihydro-4-quinolone as an off-white solid (purity 80%, yield 94%), which was used without further purification.

### 1,3-Dimethyl-1,3-dihydro-2H-imidazol-2-one

*N,N’*-dimethyl urea (1.15 g, 13 mmol) and 1.45 mL 1,2-ethanediol (26 mmol) were heated at 160°C with 0.5 g tris(triphenylphosphine)ruthenium(II) dichloride (0.5 mmol) in 10 mL diglyme for 12 hr. The mixture was then cooled to room temperature and the solvent removed by Kugelrohr short-path vacuum distillation. Residual product was purified by flash chromatography using 20:1 DCM/NH_3_/MeOH to yield 0.55 g 1,3*-*dimethyl-1,3-dihydro-2H-imidazol-2-one (38%).

Phosphoryl chloride (1.04 mL, 11 mmol) was added to a solution of 0.39 g of the dimethylimidazole-2-one (2.2 mmol) product dissolved in 8.6 mL DMF at 0 cooled to 0°C. The brown solution obtained was warmed to room temperature and held there for 2 hr, then cooled back to 0°C and quenched by addition of saturated NaHCO_3_. The aqueous mixture was extracted repeatedly with ethyl acetate and the combined organics dried over MgSO_4_. Purification by flash chromatography in 25:1 DCM/NH_3_/MeOH yielded 0.30 g (96% yield) of the desired 4‑carbaldehyde, which was used without further purification.

**Parasitological Methods**

### **Parasite culture**

*P. falciparum* 3D7A and Dd2 strains used in this study were obtained from the Malaria Research and Reference Reagent Resource Center (MR4). Parasites were grown in human erythrocytes (obtained from a transfusion blood bank in Madrid, Spain) with Albumax II medium as previously described [10]. RPMI 1640 medium (Gibco) was supplemented with 0.5% Albumax II (Invitrogen), 2% D-sucrose (Sigma-Aldrich), 0.3% glutamine (Sigma-Aldrich), and 150 µM hypoxanthine (Sigma-Aldrich). Cultures were maintained at 37°C at an atmosphere of 5% O_2_, 5% CO_2_, and 90% N_2_.

### **XC50 determination**

Parasite growth inhibition assays and 50% inhibitory concentration (XC_50_) determinations were carried out by following standard methods using the [^3^H]hypoxanthine incorporation assay [11]*.* Briefly, cultures with 0.5% parasitemia and 2% hematocrit were incubated with the drug as described for 48 h. After this period, parasites were harvested and the amount of incorporated radiolabeled precursor was determined. Radioactivity was added 24 h before harvesting of the parasites.

### **Transformed cell assays**

The Dd2 cell line containing yeast dihydroorotate dehydrogenase (DHODH) and its parental strain were cultured as described in 4.3.1 and potency of compounds determined following procedures outlined in 4.3.2. The ScURA1 gene from *Saccharomyces cerevisiae* was amplified from genomic DNA and cloned into the pLN-14 vector. The *P. falciparum* Dd2attB strain was transfected by electroporation, and stable transfectants were selected with blasticidin. Both the Dd2attB_yeastDHODH strain and its parental strain, Dd2attB, were used in these assays to determine if parasite growth inhibition was mediated by inhibition of *Pf*DHODH following rationale previously described in Painter *et al.* [12].

### **Kill rate determination**

The *in vitro* Parasite Rate Reduction assay (PRR) was conducted as previously described [13]*.* Briefly, *P. falciparum* were exposed to test compound for 120 h at a concentration corresponding to 10 x EC50. Drug was renewed daily over the entire treatment period. Samples of parasites were taken from the treated culture at intervals (24, 48, 72, 96 and 120 h time points), drug was washed out and drug-free parasites were cultured in 96-well plates by adding fresh erythrocytes and new culture media. The number of viable parasites was determined by the serial dilution technique. Four independent serial dilutions were done with each sample to correct for experimental variation.

**REFERENCES**

1. ADMET Modeler Module of ADMET Predictor. Simulations Plus, Inc., Lancaster, CA. <http://www.simulations-plus.com>.
2. Clark RD, Daga PR (2019) Building a Quantitative Structure-Property Relationship (QSPR) Model. In: Larson RS, Oprea TI (ed.) Bioinformatics and Drug Discovery, 3rd edn. Humana Press, New York
3. Fraczkiewicz R, Lobell M, Göller AH, Krenz U, Schoenneis R, Clark RD, Hillisch A (2015) Best of Both Worlds: Combining Pharma Data and State of the Art Modeling Technology To Improve in Silico pKa Prediction. J Chem Inf Model 55:389-97
4. Lisurek M, Rupp B, Wichard J, Neuenschwander M, von Kries JP, Frank R, Rademann J, Kühne R (2010) Design of chemical libraries with potentially bioactive molecules applying a maximum common substructure concept. Molec Diversity 14:401-408
5. Bandyopadhyay D, Kreatsoulas C, Brady PG, Boyer J, He Z, Scavello G Jr., Peryea T, Jadhav A, Nguyen D-T, Guha R (2019) Scaffold-based analytics: enabling hit-to-lead decisions by visualizing chemical series linked across large datasets. J Chem Inf Model 59:4880−4892
6. Plouffe D, Brinker A, McNamara C, Henson K, Kato N, Kuhen K, Nagle A, Adrián F, Matzen JT, Anderson P, Nam TG (2008) In silico activity profiling reveals the mechanism of action of antimalarials discovered in a high-throughput screen. Proc Natl Acad Sci 105:9059-9064
7. Chen Y-H, Hung H-M, Lu CC-M, Li K-C, Tzeng C-C (2004) Synthesis and anticancer evaluation of certain indolo[2,3-b]quinolone derivatives. Bioorg Med Chem 12:6539-6546
8. Yang Z, Fathi R, Zhu Q, Cho H-J, Liu Y, Sandrasagra A, Wobbe CR (2009) 4-Thio substituted quinolone and naphthyridine compounds. US Patent US20090054477A1
9. Okubo T, Kumagi T, Nakamura T, Abe K, Amada Y, Ishizaka T, Sun X-M, Sekiguchi Y, Sasako S, Shimizu T, Nagatsuka T (2011) Aminopyrrolidine compound. US Patent US8044068B2
10. Trager W, Jensen JB (2005) Human malaria parasites in continuous culture. J Parasitol 91:484–486
11. Desjardins R E, Canfield CJ, Haynes J D, Chulay J D (1979) Quantitative assessment of antimalarial activity in vitro by a semiautomated microdilution technique. Antimicrob Agents Chemother 14:710–718
12. Painter HJ, Morrisey JM, Mather MW, Vaidya AB (2007) Specific role of mitochondrial electron transport in blood-stage Plasmodium falciparum. Nature 446:88-91
13. Sanz LM, Crespo B, De-Cózar C, Ding XC, Llergo JL, Burrows JN, García-Bustos JF, Gamo FJ (2012) P. falciparum in vitro killing rates allow to discriminate between different antimalarial mode-of-action. PLOS ONE 7:e30949
